# RBM5 Is a Male Germ Cell Splicing Factor and Is Required for Spermatid Differentiation and Male Fertility

**DOI:** 10.1371/journal.pgen.1003628

**Published:** 2013-07-25

**Authors:** Moira K. O'Bryan, Brett J. Clark, Eileen A. McLaughlin, Rebecca J. D'Sylva, Liza O'Donnell, Jacqueline A. Wilce, Jessie Sutherland, Anne E. O'Connor, Belinda Whittle, Christopher C. Goodnow, Christopher J. Ormandy, Duangporn Jamsai

**Affiliations:** 1 Department of Anatomy & Developmental Biology, Monash University, Melbourne, Australia; 2 The ARC Centre of Excellence in Biotechnology & Development, Monash University, Melbourne, Australia; 3 Priority Research Centre in Chemical Biology, The University of Newcastle, Callaghan, Australia; 4 Prince Henry's Institute, Melbourne, Australia; 5 Department of Biochemistry & Molecular Biology, Monash University, Melbourne, Australia; 6 Australian Phenomics Facility, The Australian National University, Canberra, Australia; 7 The Garvan Institute of Medical Research, Sydney, Australia; Stanford University School of Medicine, United States of America

## Abstract

Alternative splicing of precursor messenger RNA (pre-mRNA) is common in mammalian cells and enables the production of multiple gene products from a single gene, thus increasing transcriptome and proteome diversity. Disturbance of splicing regulation is associated with many human diseases; however, key splicing factors that control tissue-specific alternative splicing remain largely undefined. In an unbiased genetic screen for essential male fertility genes in the mouse, we identified the RNA binding protein RBM5 (RNA binding motif 5) as an essential regulator of haploid male germ cell pre-mRNA splicing and fertility. Mice carrying a missense mutation (R263P) in the second RNA recognition motif (RRM) of RBM5 exhibited spermatid differentiation arrest, germ cell sloughing and apoptosis, which ultimately led to azoospermia (no sperm in the ejaculate) and male sterility. Molecular modelling suggested that the R263P mutation resulted in compromised mRNA binding. Within the adult mouse testis, RBM5 localises to somatic and germ cells including spermatogonia, spermatocytes and round spermatids. Through the use of RNA pull down coupled with microarrays, we identified 11 round spermatid-expressed mRNAs as putative RBM5 targets. Importantly, the R263P mutation affected pre-mRNA splicing and resulted in a shift in the isoform ratios, or the production of novel spliced transcripts, of most targets. Microarray analysis of isolated round spermatids suggests that altered splicing of RBM5 target pre-mRNAs affected expression of genes in several pathways, including those implicated in germ cell adhesion, spermatid head shaping, and acrosome and tail formation. In summary, our findings reveal a critical role for RBM5 as a pre-mRNA splicing regulator in round spermatids and male fertility. Our findings also suggest that the second RRM of RBM5 is pivotal for appropriate pre-mRNA splicing.

## Author Summary

The production of functional spermatozoa is an extraordinarily complex process that transforms a conventional round cell into the highly specialised sperm cell. These events require the coordinated activation of thousands of genes. It is likely that this complexity contributes to the large number of idiopathic infertility cases seen in humans. In an effort to improve the field's understanding of male fertility, we used a random mutagenesis screen to produce the Joey mouse line and to conclusively define RBM5 as an essential regulator of male fertility. The Joey line carries a mutation in the *Rbm5* gene, which leads to a complete block of spermatid (haploid male germ cell) differentiation and ultimately a total loss of sperm production. Our results reveal a physiological role for RBM5 in the splicing of several spermatid-expressed mRNAs that are critical for the production of spermatozoa. This study is the first to show that RBM5, via its effects on mRNA splicing in the testis, is required for male fertility. These data improve our understanding of the regulatory networks of gene expression that control sperm production and as such may lead to the development of novel approaches to enhance or suppress fertility in men.

## Introduction

Male infertility is a major medical problem affecting at least 1 in 20 men of reproductive age globally [1]. For many men, techniques such as intra-cytoplasmic sperm injection, while not offering an infertility cure, do offer the hope of fathering children. Approximately one quarter of infertile men however, produce absolutely no sperm as determined by testis biopsy [2], and for them the chances of ever fathering a child are remote. Such a clinical presentation is further frustrated by the limited ability to precisely diagnose causes of infertility in humans [1].

The production of the male gametes, spermatozoa, is a complex and dynamic process through which a haploid highly polarised cell is produced from a diploid stem cell (spermatogonia). Spermatogenesis involves mitosis and self-renewal of spermatogonia, meiosis in spermatocytes and the differentiation of haploid spermatids (termed spermiogenesis) [3]. Several hormones and growth factors trigger these processes through complex signal transduction pathways. Each of these processes is subjected to transcriptional, post-transcriptional and post-translational control [3].

RNA binding proteins (RBPs) are important players in many aspects of gene regulation. RBPs are characterised by the presence of RNA binding domains that facilitate the binding to target mRNAs [4]. Individual RBPs often contain multiple RNA binding domains, of which the most common class is the RNA recognition motif (RRM). The RRM is composed of 80–90 residues in a ßαßßαß topology with the 4-stranded anti-parallel ß-sheets forming the RNA binding surface [4]. In addition to RNA binding domains, many RBPs contain domains implicated in nucleic acid and/or protein interaction [4]. Through protein-RNA and protein-protein interacting networks, RBPs play important roles in all aspects of post-transcriptional processing of RNAs, including pre-mRNA splicing.

Alternative splicing of pre-mRNA is prevalent in mammalian cells and occurs in at least 95% of multi-exon human transcripts [5]. Alternative splicing events that occur within protein-coding regions can lead to the generation of novel open reading frames, which permit the production of multiple protein isoforms from a single gene. Conversely, such events can result in the generation of premature termination codons, which trigger mRNA degradation by the nonsense-mediated decay (NMD) pathway [6]. Moreover, alternative splicing events that occur within *cis*-acting elements in untranslated regions can lead to pronounced effects on mRNA stability [7] and translation [8]. Thus, accurate and efficient splicing is a critical feature of the regulation of gene expression and is thought to be one of the major sources for functional diversity in complex organisms. Disturbance in the balance between normal and alternative splicing is associated with many human diseases [9]. Despite the immense significance of splicing regulation in development, key splicing factors that control tissue-specific alternative splicing remain largely undefined.

RBM5 (alias LUCA15, G15, H37) is a widely expressed RBP [10]. RBM5 contains two RRMs along with other domains implicated in nucleic acid and/or protein binding [11]. RBM5 in HeLa cells is associated with the prespliceosomal A complex [12] and interacts with several spliceosomal proteins [13]–[15]. Knockdown and overexpression studies in HeLa cells demonstrate that RBM5 functions as a splicing factor and regulates alternative splicing of apoptosis-related pre-mRNAs, including *Caspase 2* [10], *FAS* receptor and *c-FLIP* [13]; and B-lymphocyte cytidine deaminase enzyme *AID* (activation-induced cytidine deaminase) [14]. The conserved role for RBM5 as a splicing factor has been shown in its plant ortholog, SUA (suppressor of abi3-5) [16]. SUA was shown to regulate the splicing of *Abi3* (abscisic acid insensitive 3), a gene that plays a role in seed maturation in *Arabidopsis* [16]. In mammals, the physiological role of RBM5 has not been defined.

Using an unbiased mouse mutagenesis approach we have identified a male-specific sterile mouse line that carries a missense mutation within the highly conserved RRM2 of the RBM5 protein. Our data reveal a crucial *in vivo* role for RBM5 in haploid male germ cell pre-mRNA splicing and fertility.

## Results

### Male sterility in Joey mutant males is caused by a missense mutation in the *Rbm5* gene

To discover genes that are essential for male fertility, we have used an ENU (N-ethyl-N-nitrosourea) mutagenesis approach [17]–[19]. Lines that presented with male sterility at a frequency of one in four, as an indication of a recessive trait, and normal mating behaviors, as indicated by the presence of copulatory plugs, were subjected to further analysis. The “Joey” line was identified as part of these screens.

**Figure 1:**
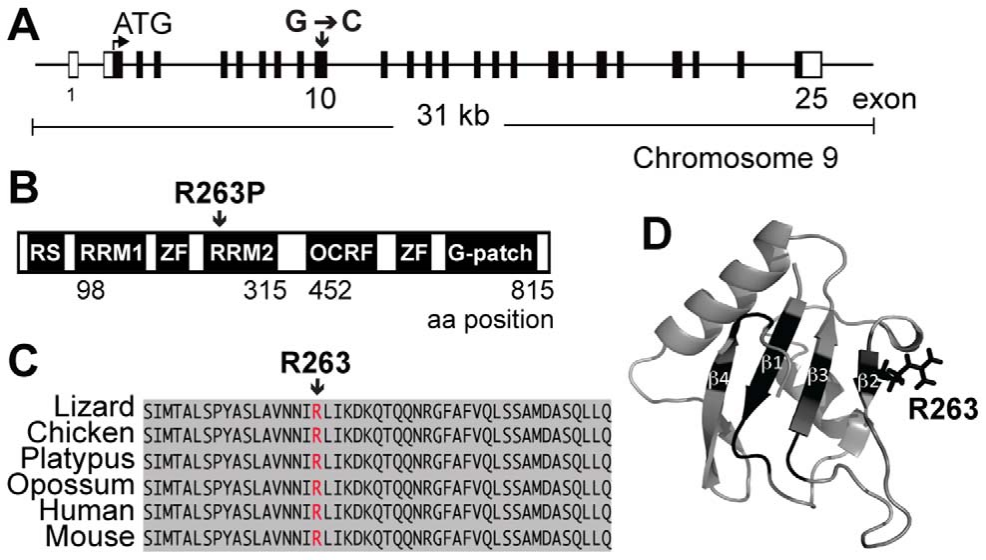
The *Rbm5^sda/sda^* results in the substitution of an arginine for proline in the RBM5 RRM2. (**A**) Schematic of the mouse *Rbm5* gene. Black bars represent exons and open bars represent untranslated regions (UTRs). (**B**) Schematic of the mouse RBM5 protein. RS: arginine-serine domain; RRM: RNA recognition motif; ZF: zinc finger motif; OCRE: octamer repeat domain; G-patch: glycine patch domain, aa: amino acid (**C**) Sequence alignment of RRM2 from different species. (**D**) Cartoon representation of the RBM5 RRM2 structure as derived using NMR (PDBid: 2LKZ) [11]. Amino acid residues within the ß2–ß4 strands that have been shown to be perturbed by RNA binding are shown in black including R263 in the ß2-strand.

The locus associated with sterility in the Joey line was mapped to a 1.6 Mb interval on chromosome 9 (rs30136959 at 106193104 bp and rs3676408 at 107831132 bp, *Ensembl* release 68), which contained 52 genes. Exons and intronic flanking regions of all 19 testis-expressed genes within the linkage interval were sequenced. A single mutational change, a G→C substitution within exon 10 of the *Rbm5* gene was identified (Figure 1A). The G→C mutation resulted in the conversion of an arginine (R) to proline (P) at position 263 (R263P) within the second RNA recognition motif (RRM2) of RBM5 (Figure 1B). Sequence alignment of RBM5 from multiple species revealed that R263 is a conserved residue within a highly conserved RRM (Figure 1C). Structural studies of RBM5 RRM2 [11] showed that R263 is positioned within the ß2-strand of the RRM that forms part of the RNA-binding interface (Figure 1D). The R263 side-chain is positioned upwards from the ß-sheet, availing itself for RNA interactions. Indeed, recent NMR studies detected perturbations to R263 upon titration of target RNA and decreased RNA binding upon the mutation of R263 to glutamic acid (E), both indicative of a direct role in RNA binding [11]. In the current study, the substitution of R263 to proline (P) would be predicted to dramatically decrease binding to target mRNAs through not only removing an interacting residue, but by disturbing the local ß2-strand secondary structure.

The homozygous mutant Joey males (hereafter referred to as *Rbm5^sda/sda^*, where sda refers to the spermatid differentiation arrest phenotype) presented with male sterility at all ages examined (*n* = 30, age 8 weeks to 6 months old). *Rbm5^sda/sda^* males were mated with wild-type (*Rbm5^WT/WT^*) or heterozygous mutant (*Rbm5^sda/WT^*) females over a period of 3 months. While normal mating behaviour, as determined by the presence of copulatory plugs, was observed, no pups were obtained from any of these breeding pairs. The sterility phenotype of the *Rbm5^sda/sda^* males was uniform on both mixed CBAxC57BL/6J and congenic C57BL/6J genetic backgrounds. Data described herein were obtained from a mixed CBAxC57BL/6J background.

To define the effect of the *Rbm5^sda/sda^* allele on female fertility, 8-week-old *Rbm5^sda/sda^* and *Rbm5^WT/WT^* females (*n* = 6 of each genotype) were mated with *Rbm5^WT/WT^* males over a period of 3 months. No difference in litter size was observed i.e. 8.2±2.1 and 8.4±2.2 pups per litter (mean±S.D.) were obtained from *Rbm5^sda/sda^* and *Rbm5^WT/WT^* female breeders, respectively. These data suggest that the *Rbm5^sda/sda^* allele had no discernable effect on female fertility.

*Rbm5^sda/sda^* mice were otherwise healthy as indicated by a body-wide pathology assessment (*n* = 10, 9 months old). Of particular note, given published associations between RBM5 and human lung cancer [20], [21], 9-month-old *Rbm5^sda/sda^* males and females had normal lung histology (data not shown).

### The consequences of RBM5 dysfunction on testis histology

**Figure 2:**
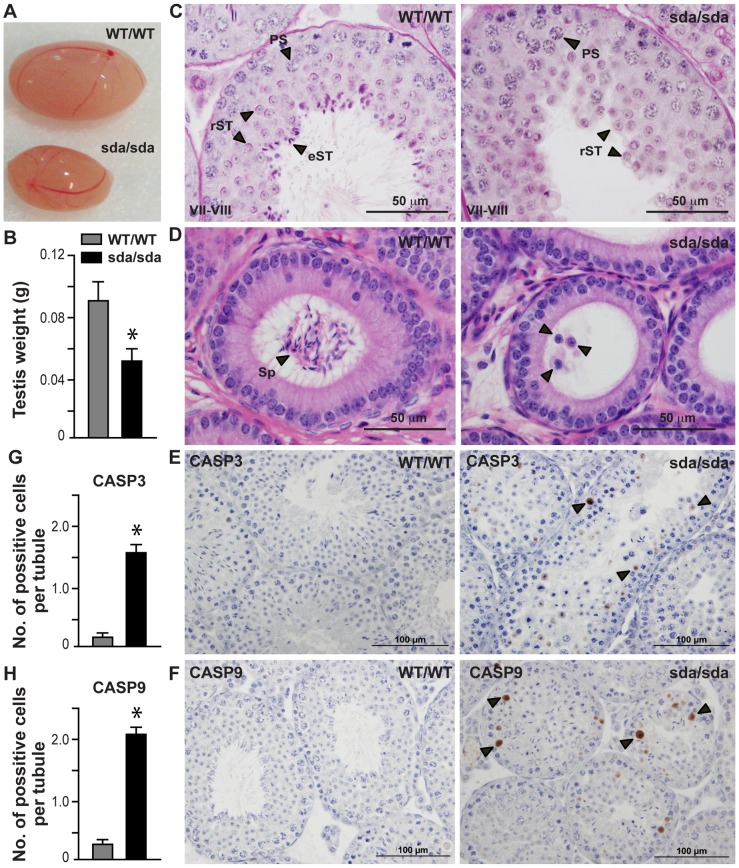
Spermatid differentiation arrest, germ cell sloughing and apoptosis lead to sterility in the *Rbm5^sda/sda^* males. (**A**) Intact testes and (**B**) testis weight of *Rbm5^WT/WT^* and *Rbm5^sda/sda^* adult mice (8-week-old). (**C**) Periodic acid-Schiff stained testis sections at stage VII–VIII and (**D**) haematoxylin and eosin stained epididymis sections of *Rbm5^WT/WT^* and *Rbm5^sda/sda^* adult mice. PS: pachytene spermatocyte, rST: round spermatid, eST: elongating and elongated spermatids; Sp: spermatozoa. **D**, arrowheads indicate sloughed immature germ cells. (**E**) Caspase 3 (CASP3) and (**F**) Caspase 9 (CASP9) immunostaining of *Rbm5^WT/WT^* and *Rbm5^sda/sda^* adult testes. Brown staining represents apoptotic cells. (**G**) Numbers of Caspase 3 and (**H**) Casapse 9 positive cells per seminiferous tubule of *Rbm5^WT/WT^* and *Rbm5^sda/sda^* adult testes. *p<0.01 (unpaired t-test).

At 8 week-of-age, the *Rbm5^sda/sda^* males displayed testicular atrophy (Figure 2A) with a ∼50% reduction in testis weight compared to *Rbm5^WT/WT^* littermates (Figure 2B). Histological analysis of testis sections revealed that spermatogenesis in the *Rbm5^sda/sda^* mice initiated successfully, and preceded up to step 8 of haploid germ cell development (Figure 2C), after which germ cells were lost via sloughing as indicated by the presence of immature germ cells within the epididymis (Figure 2D).

No spermatozoa were present in the adult *Rbm5^sda/sda^* epididymides (azoospermia) (Figure 2D). An arrest in germ cells at step 8 is significant as it is during this period of time that round spermatids are particularly responsive to the effects of hormone (androgen) withdrawal (or perturbed hormone signalling) [22], sperm tail (flagellar) development has recently initiated [23] and sperm head shaping begins through the actions of the manchette [24] and through the exchange of histones for protamines [25].

In order to explore potential for perturbed hormone signalling we measured circulating testosterone, FSH and LH levels in *Rbm5^sda/sda^* and *Rbm5^WT/WT^* littermates. No significant changes were observed (Table 1). These data suggest that the spermatid differentiation arrest phenotype was not the consequence of disturbed hormone production.

**Table 1:** Levels of serum testosterone, FSH and LH in adult *Rbm5^sda/sda^* and *Rbm5^WT/WT^* mice.

Hormone	*Rbm5^WT/WT^*	*Rbm5^sda/sda^*	p value
Testosterone (ng/ml)	0.26±0.14	0.20±0.11	0.36
FSH (ng/ml)	9.95±1.12	10.27±0.64	0.29
LH (ng/ml)	0.49±0.70	0.87±1.40	0.59

Data are expressed as mean±S.D. (standard deviation). *n* = 5 per group, age 10–14 weeks old. p values<0.05 were defined as being statistically significant (unpaired t-test, two-tailed).

Staining of *Rbm5^sda/sda^* testes and *Rbm5^WT/WT^* testis sections for cleaved Caspase 3 and Caspase 9 immunostaining indicated that significant numbers of *Rbm5^sda/sda^* germ cells were also lost via apoptosis (Figures 2E–F). Quantitative analysis of the number of Caspase-positive cells per tubule showed a significant increase in Caspase 3 (Figure 2G) and Caspase 9 (Figure 2H) positive cells in *Rbm5^sda/sda^* testes compared to *Rbm5^WT/WT^* testes (*n* = 6 mice per group, 250 tubules per mouse were counted). These data suggest that germ cells were lost by both apoptosis and sloughing. Cumulatively these data show that *Rbm5^sda/sda^* mice are sterile because of an inability to produce functional mature sperm.

### The R263P mutation generates a loss-of-function allele

**Figure 3:**
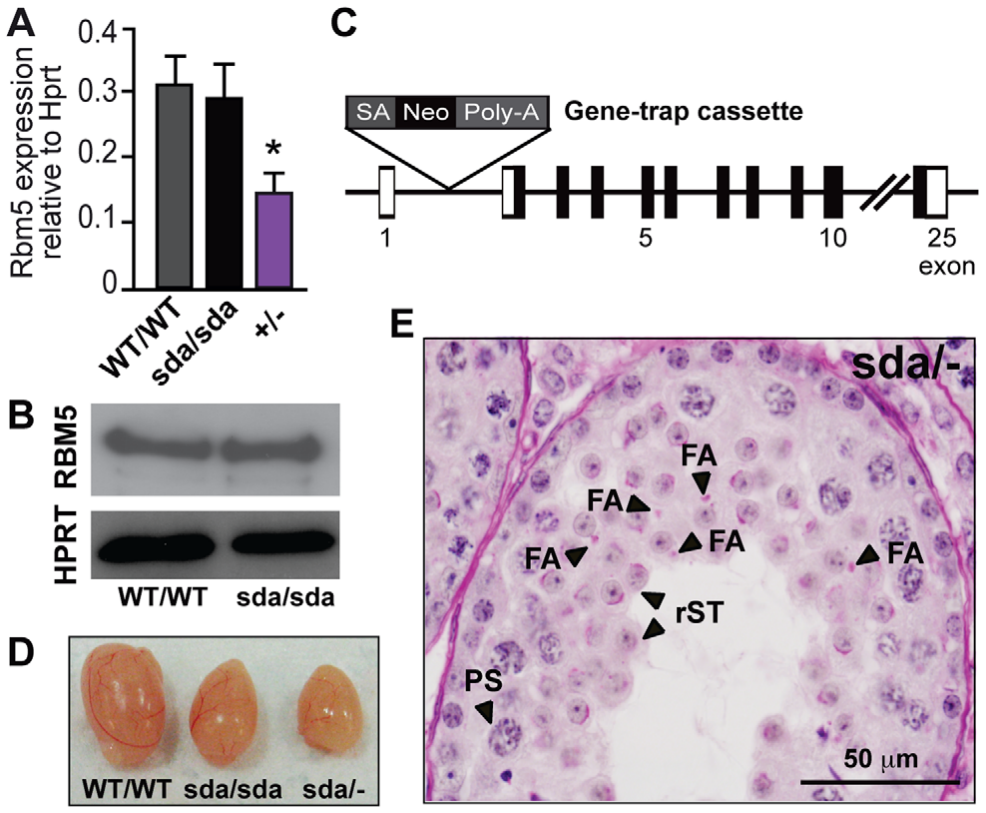
The R263 mutation results in the generation of a loss-of-function allele. (**A**) *Rbm5* mRNA expression in the *Rbm5^WT/WT^*, *Rbm5^sda/sda^* and *Rbm5^+/−^* (heterozygous knockout) postnatal day 28 testes, as determined by qRT-PCR analysis and normalised to *Hprt* expression. (**B**) RBM5 protein in postnatal day 28 testes of the *Rbm5^WT/WT^* and *Rbm5^sda/sda^* mice. HPRT was used as loading control. (**C**) Schematic of the *Rbm5* gene-trap knockout allele. SA: splice acceptor site, Neo: neomycin resistant marker, Poly-A: polyadenylation signal (**D**) Testicular atrophy in the *Rbm5^sda/−^* (compound heterozygous) and *Rbm5^sda/sda^* males compared to an *Rbm5^WT/WT^* littermate. (**E**) Periodic acid-Schiff stained testis section of a 10-week-old adult *Rbm5^sda/−^* male. PS: pachytene spermatocyte, rST: round spermatid; FA: fragmented acrosomes.

To determine whether the *Rbm5^sda/sda^* allele had an effect on *Rbm5* mRNA stability, we performed quantitative RT-PCR (qRT-PCR) using postnatal day 28 total testis RNA i.e. in accordance with the germ cell arrest observed in *Rbm5^sda/sda^* testes. No significant difference in the relative abundance of *Rbm5* mRNA was observed in the *Rbm5^sda/sda^* compared to *Rbm5^WT/WT^* testes (Figure 3A). Similarly, the mutant RBM5 protein in the *Rbm5^sda/sda^* postnatal day 28 testis was detected at similar levels to that of *Rbm5^WT/WT^* testis (Figure 3B). These data suggest that the sterility phenotype was not due to a reduction in mRNA or protein production.

To formally confirm that the *Rbm5^sda/sda^* allele was responsible for male-specific sterility phenotype, we utilised a genetic complementation approach. We generated mice carrying a heterozygous *Rbm5* knockout allele (referred to as *Rbm5* ^+/−^) using a gene-trap ES cell line that carried a transcriptional terminator cassette within intron 1 of the *Rbm5* gene (Figure 3C). The level of *Rbm5* transcript in the *Rbm5* ^+/−^ postnatal day 28 testis, as determined by qRT-PCR analysis, was reduced by ∼50% compared to *Rbm5^WT/WT^* and *Rbm5^sda/sda^* testes (Figure 3A). We mated the *Rbm*5^+/−^ males with *Rbm5^sda/sda^* females and the *Rbm5^+/−^* females with the *Rbm5^sda/WT^* males to produce compound heterozygous offspring (referred to as *Rbm5^sda/−^* ^)^. To test their fertility status, 8–10 week-old *Rbm5^sda/−^* males (*n* = 10) were bred with *Rbm5^WT/WT^* females over a 6-month period. *Rbm5^sda/−^* males were sterile. Detailed phenotypic analysis revealed that the *Rbm5^sda/−^* males displayed testicular atrophy (Figure 3D) and spermatid differentiation arrest comparable to the *Rbm5^sda/sda^* males (Figure 3E). The only observed difference between *Rbm5^sda/sda^* and *Rbm5^sda/−^* testis histology were pronounced acrosome defects in *Rbm5^sda/−^* sections (Figure 3E). Test breeding of *Rbm5^sda/−^* females at 8–10 week-of-age (*n* = 10) with *Rbm5^WT/WT^* males indicated that they were fertile i.e. 6–10 pups per litter. These results further confirm that the *Rbm5^sda/sda^* allele is the cause of male-specific sterility in the *Rbm5^sda/sda^* mouse line, and strongly suggest that the R263P mutation generates a loss-of-function allele. The exacerbation of the acrosome phenotype between *Rbm5^sda/sda^* and *Rbm5^sda/−^*, however, suggests that the *Rbm5^sda^* allele may retain some functional activity. A likely role for RBM5 in acrosome development is described below.

### RBM5 is ubiquitously expressed but highly enriched in the testis

**Figure 4:**
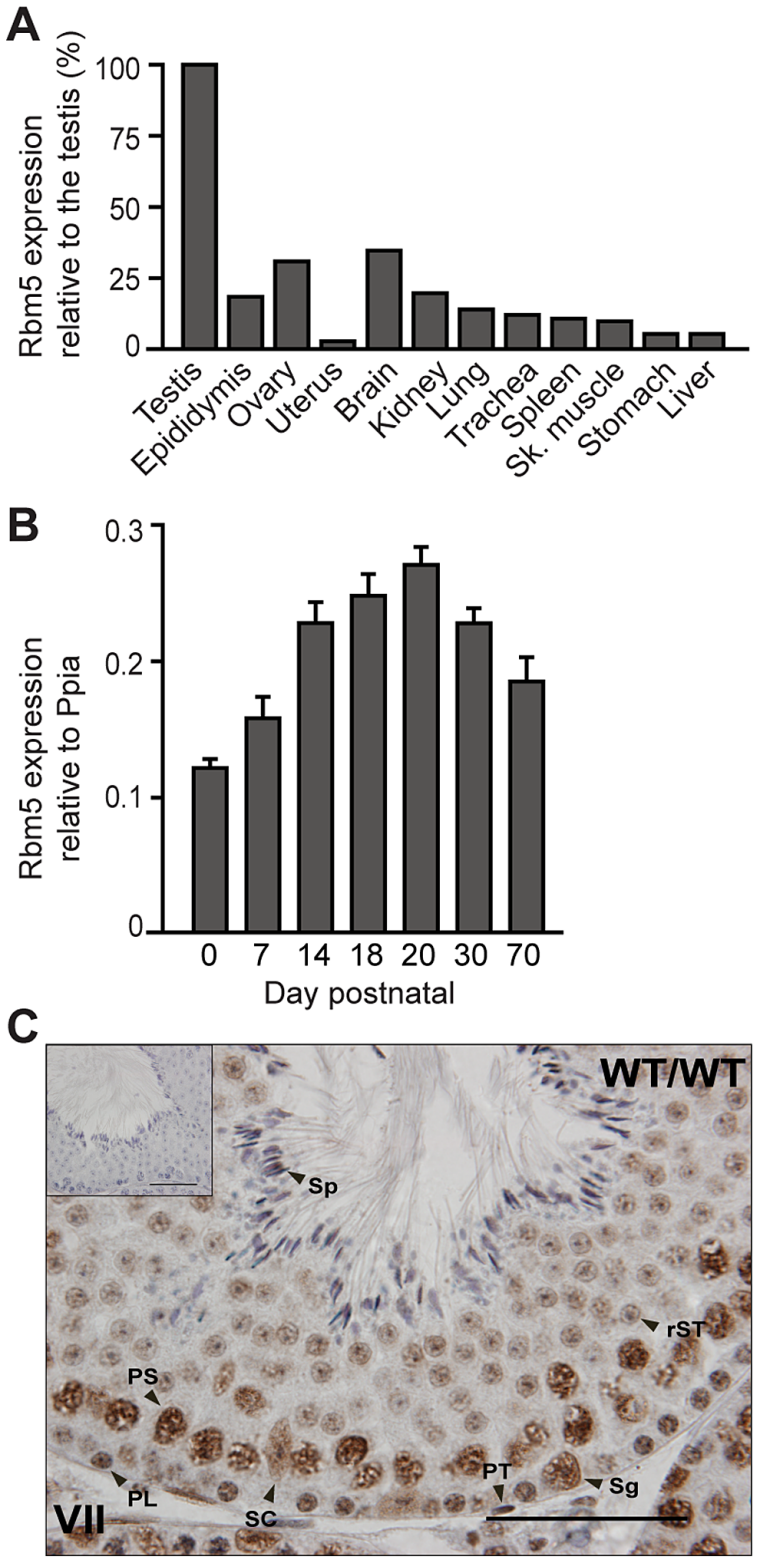
*Rbm5* mRNA is highly expressed in the testis where the protein localises in somatic and germ cells. (**A**) *Rbm5* mRNA expression in various tissues and (**B**) during the establishment of the first wave of spermatogenesis. Sk: skeletal. Error bars = S.D. (standard deviation, *n* = 3 wild-type C57BL/6JxCBA mice, per age group) (**C**) RBM5 protein localisation in an adult *Rbm5^WT/WT^* testis. (**C, inset**) RBM5 antibody pre-absorption control. Sg: Type A spermatogonia, PL: preleptotene spermatocyte, PS: pachytene spermatocyte, rST: round spermatid, Sp: spermatozoa, SC: Sertoli cell, PT: peritubular cell. Scale bars = 50 µm.

Consistent with public gene expression databases, we detected *Rbm5* mRNA expression in many adult mouse tissues but with the strongest expression in the testis (Figure 4A). A survey of testicular *Rbm5* mRNA expression at different time points during the establishment of spermatogenesis revealed expression in neonate through to adult testes, suggesting its expression in multiple cell types. Levels of expression were enriched in postnatal day 14 through to day 30, (Figure 4B), suggesting that *Rbm5* was most highly expressed in spermatocytes and round spermatids. Consistently, RBM5 protein was found in germ cells and somatic cells of the testis i.e. Sertoli and peritubular cells (Figure 4C). In germ cells, RBM5 localised to the nucleus and cytoplasm of type A spermatogonia, spermatocytes and round spermatids (Figure 4C). Testis sections interrogated with pre-absorbed RBM5 antibody did not show any positive staining (Figure 4C, insert).

### The R263P mutation affects the expression of genes in several pathways including those implicated in germ cell adhesion, spermatid head shaping, and acrosome and tail formation

The complete arrest of the spermatogenic cycle during spermatid differentiation was the most dramatic phenotype observed in the *Rbm5^sda/sda^* mice and the ultimate cause of sterility. As such the function of RBM5 in spermatids is the focus of this study. In order to investigate the effects of the R263P mutation on the round spermatid transcriptional program, round spermatids were isolated from postnatal day 28 *Rbm5^sda/sda^* and *Rbm5^WT/WT^* mice and subjected to microarray analysis using Illumina mouse WG6v2 arrays. A total of 483 probe sets out of 45,275 probe sets showed a >2-fold change in *Rbm5^sda/sda^* compared to *Rbm5^WT/WT^* round spermatids. 263 probes (234 genes) were increased and 220 probes (208 genes) were decreased (Supplementary Table S1). These probe sets were submitted to the DAVID Functional Clustering algorithm (http://david.abcc.ncifcrf.gov) [26], [27] in order to elucidate biological functions that were significantly enriched in the differentially expressed genes. The top most significant functions associated with differentially expressed genes in *Rbm5^sda/sda^* round spermatids were actin-binding, microtubule cytoskeleton and endocytosis (Table 2).

**Table 2:** Functional clustering of differentially expressed genes identified by microarray analysis of RNAs isolated from round spermatids of *Rbm5^sda/sda^* and *Rbm5^WT/WT^* postnatal day 28 testes.

Function	Enrichment score of cluster	p value	aDifferentially expressed genes
Actin-binding	1.97	5.80E-03	*Cap1*, *Tmod4*, *Lasp1*, *LOC100044756*, *Arpc*3, *Clmn*, *Emd*, *Fscn3*, *Mprip*, *Spnb1*
Microtubule cytoskeleton	1.95	4.20E-03	*Arl3*, *6330503K22Rik*, *Anxa11*, *Dctn6*, *Emd*, *Kif23*, *Rps6ka1*, *Tubb6*
Endocytosis	1.56	3.8E-02	*Cap1*, *Msr1*, *Ppt1*, *Picalm*, *Fcgr3*, *Gata2*, *Lrp11*, *Sgca*, *Ndel1*

a Differentially expressed genes associated with a particular significant function are given.

During spermiogenesis, actin-containing filaments and microtubules are involved in multiple aspects of development i.e. the ectoplasmic specialisations (ESs), acroplaxome and the manchette [28]. ESs are required for germ cell attachment to Sertoli cells and germ cell movement, orientation and detachment (spermiation). The actin-containing acroplaxome is an adhesion point of acrosomal formation and cooperates with the microtubule-based manchette to facilitate nuclear shaping [29], [30]. In addition, some vesicles and molecules required for tail formation are delivered via the intra-manchette transport [31]. Perturbation of the expression of genes associated with actin-binding and the microtubule network in *Rbm5^sda/sda^* round spermatids raises the possibility that (i) the failure in spermatid head shaping and pre-mature spermatid detachment (sloughing) were caused by abnormalities in spermatid actin filaments and adhesion to Sertoli cells, (ii) the failure in sperm head shaping and tail formation were caused by abnormalities in the microtubule cytoskeleton network, and (iii) the severe acrosomal defects in *Rbm5^sda/−^* mice may be associated with abnormal actin in the acroplaxome. Moreover, RBM5 dysfunction also affected the expression of genes associated with the endocytosis pathway (Table 2). The precise role of endocytosis in spermatogenesis remains largely unknown, however endocytosis in spermatids has been proposed as one of the mechanisms that regulate the uptake of macromolecules and vesicle-containing acrosomal enzymes required for fertilisation [32].

In summary, these data suggest that RBM5 dysfunction affects expression of genes in several pathways including those implicated in germ cell adhesion, spermatid head shaping, and acrosome and tail formation.

### RBM5 interacts with splicing factors SFPQ and hnRNP A2/B1 and in round spermatids

In order to define the pathway(s) RBM5 is involved in, we first exploited immunoprecipitation to isolate RBM5 and its associated proteins from purified round spermatid extracts. Recovered immunocomplexes were analysed by mass spectrometry. We identified 40 putative RBM5 binding partners (Supplementary Table S2). Consistent with findings from human cell lines [12]–[15], immunoprecipitation data from spermatids revealed that many RBM5 putative binding proteins have been implicated in pre-mRNA splicing. These included four hnRNPs (heterogeneous nuclear ribonucleoproteins) i.e. hnRNP A2/B1, hnRNP K, hnRNP M, and hnRNP UL1; two SR proteins, SFRS1 (serine/arginine-rich splicing factor 1, alias ASF and SF2) and PSIP1 (PC4 and SFRS1-interacting protein); splicing factor SFPQ (proline- and glutamine-rich); RNA helicase DDX5 (DEAD (Asp-Glu-Ala-Asp) box helicase 5) and U1A (small nuclear ribonucleoprotein polypeptide A). In addition to splicing factors, several RBPs that play a role in different aspects of RNA biogenesis were also identified as RBM5 putative binding partners (Supplementary Table S2). These included PABP1 (polyadenylate-binding protein 1), DDX4 (DEAD (Asp-Glu-Ala-Asp) box helicase 4, alias MVH and VASA), PSPC1 (paraspeckle component 1) and ELAV1 (embryonic lethal abnormal vision-like protein 1, alias HuR). These data suggest that RBM5 plays a role in RNA processing through a network of protein binding partners.

**Figure 5:**
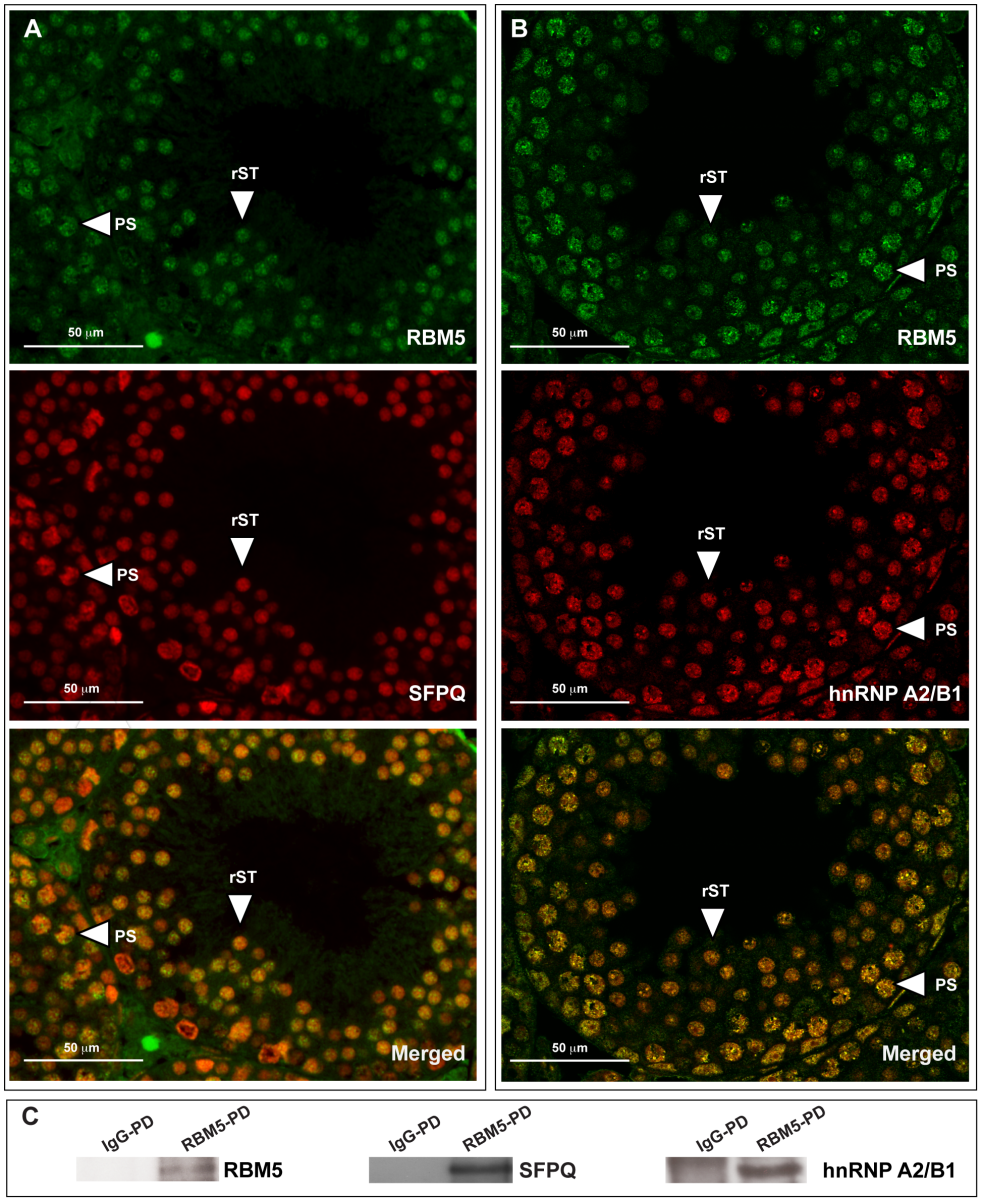
RBM5 co-localises and interacts with splicing factors hnRNP A2/B1 and SFPQ in male germ cells. (**A**) RBM5 co-localises with SFPQ and (**B**) hnRNP A2/B1 in postnatal day 28 *Rbm5^WT/WT^* testis. PS: pachytene spermatocyte, rST: round spermatid. (**C**) RBM5 pull down confirms RBM5-SFPQ and RBM5-hnRNP A2/B1 interaction in round spermatids. IgG-PD: pull down using IgG, RBM5-PD: pull down using the RBM5 antibody.

As RBM5 has been shown to play a role in splicing regulation in human cell lines [12]–[15] and plants [16], the focus in this study is to define whether its splicing function is conserved in male germ cells. Two enriched putative binding proteins (based on Mascot scores and number of matched peptides, Supplementary Table S2) with strong links to splicing regulation, SFPQ and hnRNP A2/B, were chosen for further analysis. Within the adult mouse testis, both proteins localised strongly in the nucleus of spermatocytes and round spermatids and co-localised with RBM5 (Figure 5A). Further, we confirmed that RBM5 interacted with SFPQ and hnRNP A2/B1 as determined by co-immunoprecipitation of round spermatid extracts (Figure 5B and Supplementary Figure S1). Collectively these results suggest a conserved role for RBM5 as a splicing regulator in haploid male germ cells.

### Identification of RBM5 target mRNAs in round spermatids

RBM5 target mRNAs have not been identified in male germ cells. We therefore adapted the RBM5 immunoprecipitation and coupled it to a microarray to identify RBM5 target mRNAs in purified round spermatids. Three independent pull down sets from three biological replicates were performed and the level of each target was averaged. Enrichment scores (fold enrichment) were defined by the average level of each target in the pull down set versus their level of expression in the total round spermatid extracts (input material). Targets with ≥2-fold enrichment were defined as RBM5 targets. Using these selection criteria, we identified 11 RBM5 putative targets. The three most highly enriched targets (enriched scores ≥2.5 fold) were *St5* (suppression of tumorigenicity 5), *Asb1* (ankyrin repeat and SOCS box-containing 1) and *Pla2g10* (phospholipase A2, group X) (Table 3). Bioinformatics analysis using the Ingenuity Pathway Analysis (IPA) software suggested that RBM5 targets participate in diverse biochemical pathways (Table 3). Of note, *Asb1*, *Plag2g10*, *Kif17* (Kinesin family member 17) and *Tagap1* (T-cell activation GTPase activating protein 1) have been linked to spermatogenesis, in particular during haploid germ cell development [33]–[38].

**Table 3:** RBM5 target mRNAs in round spermatids identified using RNA pull down coupled with microarrays.

mRNA	Full name	Gene ID	Fold enrichment	Proposed function	Reference(s)
*St5*	suppression of tumorigenicity 5	ENSMUSG00000031024	3.1	MAPK/ERK signalling	[44]–[46]
*Asb1*	ankyrin repeat and SOCS box-containing 1	ENSMUSG00000026311	2.8	JAK/STAT signalling, involved in spermatogenesis	[47]
*Pla2g10*	Phospholipase A2, group X	ENSMUSG00000022683	2.6	Sperm maturation and motility regulator	[34], [35]
*Kif17*	Kinesin family member 17	ENSMUSG00000028758	2.4	Sperm tail protein, involved in anterograde intraflagellar transport (IFT) and cilium biogenesis.	[36], [37]
*Anks3*	Ankyrin repeat and sterile alpha motif domain containing 3	ENSMUSG00000022515	2.4	Unknown	
*Rangap1*	RAN GTPase activating protein 1	ENSMUSG00000022391	2.4	RAN GTP/GDP cycle regulator, involved in mRNA processing and transport	[63]
*Tagap1*	T-cell activation GTPase activating protein 1	ENSMUSG00000052031	2.3	T-complex distorter (Tcd), involved in sperm tail movement	[38]
*Nfx1*	Nuclear RNA export factor 1	ENSMUSG00000010097	2.3	Transcriptional repressor	[64]
*Hhatl*	Hedgehog acyltransferase-like	ENSMUSG00000032523	2.1	Unknown	
*Cftr*	Cystic fibrosis transmembrane conductance regulator homolog	ENSMUSG00000041301	2.1	Transportation of chloride and thiocyanate ions across epithelial cell membranes. Mutations of the CFTR gene leads to cystic fibrosis and congenital absence of the vas deferens.	[65]
*2700094K13Rik*	RIKEN cDNA 2700094K13	ENSMUSG00000076437	2.1	Unknown	

**Figure 6:**
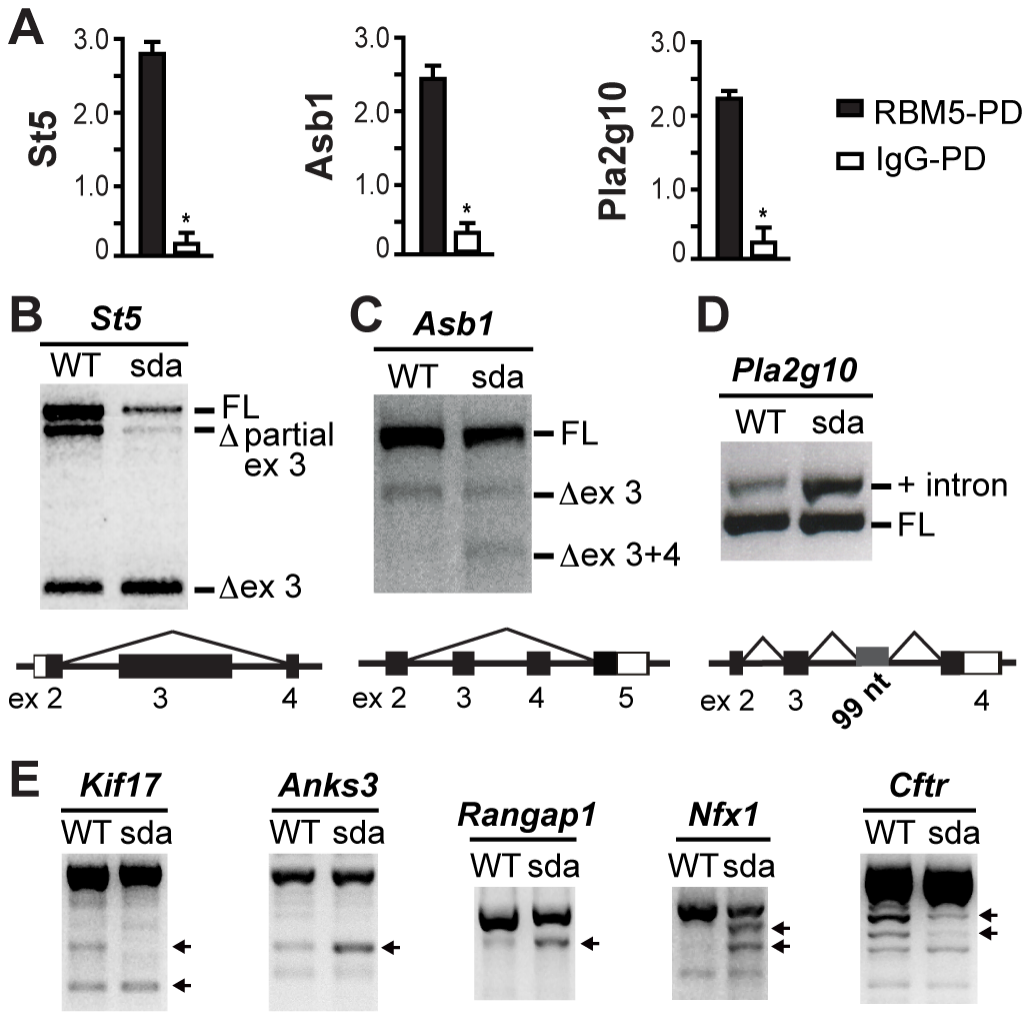
RBM5 RRM2 is required for appropriate pre-mRNA splicing. (**A**) Relative abundance of *St5*, *Asb1* and *Plg2g10* is significantly increased in the RBM5 pulled down (RBM5-PD) samples compared to that of IgG-PD controls. Error bars = S.D. (standard deviation, *n* = 3 sets of each pull down). * indicates statistical significance (p<0.05, t-test). (**B–D**) The R263P mutation in the RRM2 of RBM5 leads to aberrant splicing of *St5*, *Asb1* and *Pla2g10*. Black bars represent exons and open bars represent UTRs. FL: full-length, ex: exon, Δ ex 3 refers to exon 3 skipping, Δ ex 3+ex 4 refers to exon 3 and exon 4 skipping, and + intron refers to intron retention. (**E**) Splicing defects of *Kif17*, *Anks3*, *Rangap1*, *Nfx1* and *Cftr*. Transcripts with obvious shift in their relative intensities are indicated by arrows.

To further verify the specificity of the RNA pull down experiments, qRT-PCR was employed to determine the relative abundance of *St5*, *Asb1* and *Pla2g10* in the RBM5-pulled down and IgG-pulled down control samples. All three targets showed a significant increase in their relative abundance in the RBM5-pulled down samples compared to that of IgG-pulled down samples (Figure 6A), thus confirming the specificity of our pull down experiments.

### RBM5 is a splicing factor in round spermatids and its RRM2 is required for appropriate splicing of its target pre-mRNAs during spermiogenesis

Genetic complementation and molecular modelling results suggest that the R263P mutation resulted in a loss-of-function allele. In HeLa, B-lymphocyte and plant cells, RBM5 regulates the splicing of target pre-mRNAs [10], [13], [14], [16]. As the testis is a tissue that contains high levels of alternative splicing [39], we first asked whether the splicing function of RBM5 is conserved in haploid male germ cells and then, if this function was perturbed by the R263P mutation. We used RT-PCR analysis to assess the splicing pattern of RBM5 putative targets in round spermatids of the *Rbm5^WT/WT^* and *Rbm5^sda/sda^* males. First, we analysed the three most highly enriched targets *St5*, *Asb1* and *Pla2g10*. Consistent with our hypothesis, the splicing of these three targets was altered in the *Rbm5^sda/sda^* round spermatids (Figures 6B–D). We note that based on microarray data (Supplementary Table S1), the total expression level of all RBM5 target mRNAs was unchanged between *Rbm5^sda/sda^* and *Rbm5^W/WT^* round spermatids. These data suggest that RBM5 is specifically involved in regulating splicing of these targets rather than their stability, turnover or translation. These data do not however, preclude that RBM5 has a role in determining the mRNA stability, rates of mRNA turnover and/or translation of other transcripts. Such a function would be supported by the binding of RBM5 to PABP1 [40], ELAVL1 [41], DDX4 [42], and MATR3 [43] (Supplementary Table S2). Such a role for RBM5 will be explored in future studies.

ST5 has been proposed to play a role in a MAPK/ERK signalling in COS-7 and pancreatic ß cell lines [44]–[46]. The role for ST5 in spermatogenesis is unknown. Based on the *Ensembl* database (release 68), the mouse *St5* gene is predicted to give rise to 4 transcripts (ENSMUST00000077909, ENSMUST00000079282, ENSMUST00000084738 and ENSMUST00000168005) via alternative splicing and the use of an alternative promoter. Within round spermatids from both the *Rbm5^WT/WT^* and *Rbm5^sda/sda^* mice, we detected three *St5* transcripts including the ENSMUST00000077909 (full-length), a novel transcript with partial exon 3 skipping, and the ENSMUST00000084738 transcript within which the entire of exon 3 was skipped (Figure 6B). As illustrated the R263P mutation enhanced exon 3 skipping thus reducing the level of full-length transcript in the *Rbm5^sda/sda^* round spermatids (Figure 6B).

ASB1 is a member of the suppressor of cytokine signalling (SOCS) protein family. SOCS proteins have been implicated in the regulation of the JAK/STAT signalling pathway [47]. The lack of ASB1 in mice results in hypospermatogenesis and a progressive loss of germ cells [33]. Based on the *Ensembl* database, the mouse *Asb1* gene is predicted to give rise to two transcripts (ENSMUST00000027538 and ENSMUST00000086843) via the use of an alternative promoter. Within the *Rbm5^WT/WT^* round spermatids, we detected two transcripts i.e. the ENSMUST00000027538 (full-length) and a novel transcript wherein the entire of exon 3 was skipped (Figure 6C). Analogous to *St5*, the R263P mutation stimulated the skipping of both exon 3 and 4 in the *Rbm5^sda/sda^* round spermatids (Figure 6C). Based on sequence analysis, the exons 3 and 4 skipped transcript is predicted to encode a novel ASB1 protein isoform.

PLA2G10 is a phospholipase enzyme in the acrosome of spermatozoa that is released during the acrosome reaction and has the ability to modulate the motility of capacitated sperm [34], [35]. Based on the *Ensembl* database, the mouse *Pla2g10* gene is predicted to give rise to four transcripts (ENSMUST00000023364, ENSMUST00000115807, ENSMUST00000127780 and ENSMUST00000156504) via alternative splicing and the use of an alternative promoter. Within the *Rbm5^WT/WT^* and *Rbm5^sda/sda^* round spermatids, we detected the ENSMUST00000023364 (full-length) transcript and a novel spliced variant transcript that retained 99 nucleotides from intron 3 (Figure 6D). The R263P mutation resulted in a shift in the balance of transcripts towards the latter variant containing the intron 3 sequence (Figure 6D). The retention of the intronic sequence results in the introduction of a premature stop codon.

In order to determine if altered splicing is broadly associated with RBM5 dysfunction we defined the splicing pattern of the remaining eight putative RBM5 targets using RT-PCR. As shown in Figure 6E, the *Rbm5^sda/sda^* round spermatids contained aberrant splicing of *Kif17* (Kinesin family member 17), *Anks3* (Ankyrin repeat and sterile alpha motif domain containing 3), *Rangap1* (RAN GTPase activating protein 1), *Nfx1* (Nuclear RNA export factor 1) and *Cftr* (cystic fibrosis transmembrane conductance regulator homolog). We did not detect splicing defects in *Tagap1*, *Hhatl* and *2700094K13Rik* (data not shown). Together, our data demonstrate for the first time that RBM5 functions as a critical splicing factor that tightly regulates the balance between normal and alternative splicing of several pre-mRNAs including those with functions that have been linked to haploid male germ cell development and male fertility. Our results also suggest that the RRM2 of RBM5 is pivotal for appropriate pre-mRNA splicing.

### An example of the consequences of dysfunctional RBM5-mediated splicing: *St5* and ERK1/2 activation

In order to assess the consequence of splicing defects, we chose to further analyse the most highly enriched RBM5 target, *St5*. *St5* is of particular interest due to its proposed role in regulating MAPK/ERK signalling in somatic cells [44]–[46]. MAPK signalling has been implicated in cell growth, differentiation and apoptosis in many biological systems, and as such various established tools are available to evaluate the activation of the pathway. Importantly, the MAPK pathway is involved in many aspects of spermatogenesis, post-testicular sperm maturation and fertilisation [48]. Studies in rat models indicated that ERK1/2 activity is required for germ cell-Sertoli cell adhesion [49], [50]. Increased levels of activated ERK1/2 (via chemical treatments) in the rat testis is associated with germ cell loss [49], [50]. These findings and germ cell sloughing defects in the *Rbm5^sda/sda^* mice (Figure 2D) prompted us to further investigate the consequence of *St5* splicing defects on MAPK/ERK signalling.

**Figure 7:**
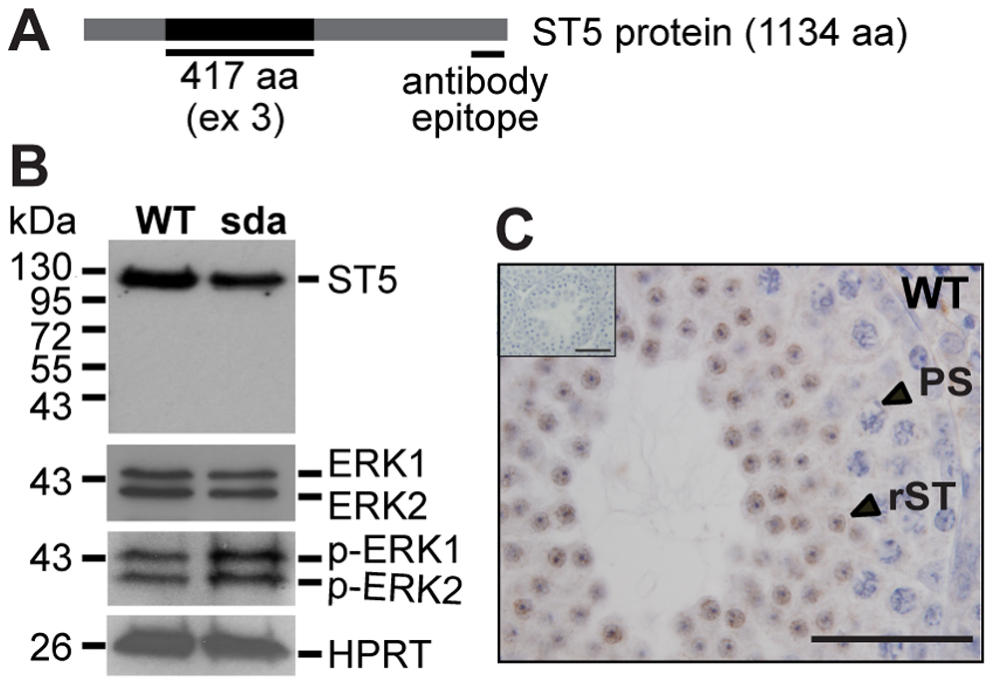
ST5 negatively regulates ERK signalling in round spermatids. (A) Schematic of the mouse ST5 protein. (B) Reduction of the ST5 protein leads to hyperactivation of ERK1/2 in round spermatids. HPRT was used a loading control. (C) ST5 localises strongly in the cytoplasm of round spermatids as determined by immunohistochemistry of postnatal day 28 testis section. (C, insert) No positive signal was observed when the ST5 antibody was omitted. Scale bars = 50 µM.

In humans, the *ST5* gene (ENSG00000166444) is predicted to give rise to 61 transcripts derived from alternative splicing or the use of alternative promoters. Of these, the 126 kDa full-length isoform (ENSP00000433528) has been shown to positively regulate ERK2 phosphorylation in COS-7 cells [44], [45], and ERK1 and ERK2 phosphorylation in the pancreatic ß cell line MIN6 [46], in response to epidermal growth factor (EGF) stimulation. Thus we asked if ERK signalling is affected in the *Rbm5^sda/sda^* round spermatids. First, the effect of altered splicing on the levels of ST5 protein was determined using immunoblotting. Exon 3 skipping in the ENSMUST00000084738 transcript was predicted to result in an in-frame deletion of 417 amino acids near the N-terminus of the protein (Figure 7A), producing a protein with a predicted molecular weight of 82 kDa. Using an ST5 antibody that recognized the C-terminal region (Figure 7A) present in all of the predicted mouse *St5* transcripts, only the 126 kDa isoform (ENSMUSP00000077067) was detected in both *Rbm5^WT/WT^* and *Rbm5^sda/sda^* round spermatids (Figure 7B). The 126 kDa isoform was significantly down-regulated in *Rbm5^sda/sda^* spermatids (Figure 7B). This result indicates that the 126 kDa isoform is the only isoform of ST5 protein produced in round spermatids, and alternatively spliced transcripts do not give rise to new protein isoforms and/or the new isoforms are highly unstable and rapidly degraded. Thus the *Rbm5^sda/sda^* allele results in a significant decrease in ST5 full-length protein. Furthermore, we showed that ST5 localised strongly to round spermatids (Figure 7C), suggesting a role for ST5 in spermiogenesis.

In order to monitor the effect of decreased ST5 bioavailability on ERK1/2 activation we determined the total ERK1/2 and phosphorylated ERK1/2 (p-ERK1/2), as markers of activation, using immunoblotting. *Rbm5^WT/WT^* and *Rbm5^sda/sda^* round spermatids contained similar levels of total ERK1/2 proteins, however, p-ERK1/2 levels were significantly increased in *Rbm5^sda/sda^* round spermatids compared to those from the *Rbm5^WT/WT^* males (Figure 7B). These data reveal that RBM5 has a critical role in the splicing of *St5* pre-mRNAs, and an alteration in the balance between the normal and alternative splicing is an important factor that regulates MAPK/ERK signalling output during spermatid differentiation.

While the example of *St5* is compelling and may indeed contribute to the premature germ cell sloughing phenotype observed *Rbm5^sda/sda^* males, members of numerous and apparently separate pathways were identified as being RBM5 target mRNAs. As such it is most likely that the *Rbm5^sda/sda^* phenotype was caused by the parallel disruption of multiple pathways involved in spermatid differentiation.

## Discussion

### RBM5 is absolutely required for appropriate pre-mRNA splicing in round spermatids and male fertility

Our findings demonstrate that RBM5 is an essential splicing factor in round spermatids *in vivo*. Our data suggests that RBM5 acts through a protein-protein interaction network that includes several SR and hnRNP proteins to control the splicing of its target pre-mRNAs, including those required for spermatid differentiation. Deficits in RBM5 function, as exemplified in the *Rbm5^sda/sda^* males, result in male sterility characterised by a block during haploid germ cell development.

Genetic complementation data suggests that the R263P mutation resulted in a loss-of-function allele. This mutation occurs at the RNA binding interface of the RRM2 of RBM5 and would be expected to impact negatively on the ability of RBM5 to interact with target mRNAs. From the current general understanding of RNA binding by RRMs [51] and through specific structural studies of RBM5 RRM2 [11], R263 potentially underlies both the affinity and specificity of the RNA interaction. A proline mutation, in particular, would abrogate this interaction and, furthermore, would disturb the local ß2-strand secondary structure and thus potentially further decrease the affinity of the interaction of RBM5 with target mRNAs.

Cumulatively our pre-mRNA splicing analyses suggest that RBM5 functions to promote exon inclusion in the majority of cases. There were however, three different types of splicing patterns observed in *Rbm5^sda/sda^* round spermatids: (i) exon skipping which altered the ratio between full-length mRNA and shorter spliced transcripts (as seen in *St5*, *Kif17*, *Anks3*, *Rangap1* and *Cftr*); (ii) increased intron retention (as seen in *Pla2g10*) which also altered transcript ratios, and (iii) the production of novel spliced transcripts (as seen in *Asb1* and *Nfx1*). It should be acknowledged that a shift in the stability of particular transcripts in some of these examples could explain the altered transcript ratios. This possibility is not however supported by the bulk of the surrounding data. For example, if the R263P mutation resulted in major effects on the stability and/or pre-mature translation of RBM5 target mRNAs, it would be expected that the overall levels of RBM5 target mRNAs would be significantly altered in *Rbm5^sda/sda^* round spermatids compared to *Rbm5^WT/WT^*. Based on our analyses of whole round spermatid transcriptome, the levels of the 11 RBM5 target mRNAs were not changed between *Rbm5^sda/sda^* and *Rbm5^WT/WT^*. Moreover, the detection of a novel spliced isoform of *Asb1* (exons 3 and 4 skipping) and two novel spliced isoforms of *Nfx1* which do not exist in *Rbm5^WT/WT^* round spermatids, clearly demonstrates splicing defects in the *Rbm5^sda/sda^* germ cells. These data in combination with results on the role of RBM5 in human cell lines [10], [13], [14] and in plants [16] demonstrate the conserved *in vivo* role for RBM5 as a splicing regulator across multiple cell types and species.

### Splicing defects of RBM5 targets lead to the altered expression of genes that regulate spermatid head shaping, acrosome and tail formation, and germ cell adhesion

RBM5 is ubiquitously expressed in adult tissues however, the highest expression was observed in the testis where RBM5 was localised to somatic, stem cells, meiotic and post-meiotic germ cells. The *Rbm5^sda/sda^* allele disrupted male fertility as a consequence of complete spermatogenic arrest during spermatid differentiation. During this period, round spermatids undergo a series of dramatic morphological alterations which give rise to highly polarised spermatozoa. This transformation involves chromatin reorganisation and condensation, acrosome formation, cytoplasmic removal, sperm tail assembly and spermiation (sperm release) [52]. The timing of spermatogenic arrest in the *Rbm5^sda/sda^* mice is concomitant with sperm tail development and the initiation of the condensation of the haploid nucleus. As a result of germ cell sloughing and apoptosis, no sperm were found in the reproductive tract of the *Rbm5^sda/sda^* mice. The analogous phenotype in humans is referred to as azoospermia as a consequence of germ cell arrest [1], [2].

The results from microarray analysis indicate that the R263P mutation results in an altered transcriptional program in *Rbm5^sda/sda^* round spermatids. These transcriptional changes were associated with particular functions known to be important during round spermatid development and mirrored in the *Rbm5^sda/sda^* phenotype. Significant associated transcriptional networks included actin filament and microtubule-based organisation, and endocytosis. These data suggest that a combination of these defects underlie the premature sloughing of round spermatids from Sertoli cells, the failure in sperm head shaping, and acrosome and tail formation. Cumulatively these data suggest that male sterility in *Rbm5^sda/sda^* mice is the result of the deregulation of multiple parallel pathways rather than any one specific RBM5 target.

To further demonstrate the biological consequence of splicing defects and germ cell loss phenotype, we performed a detailed analysis of the most highly enriched RBM5 target, *St5*. The R263P mutation leads to an increase in *St5* exon 3 skipping and a significant reduction of the full-length ST5 protein in *Rbm5^sda/sda^* round spermatids. Our results indicate that the two alternatively spliced *St5* transcripts do not give rise to additional ST5 protein isoforms. Thus, the regulation of alternative splicing of *St5* pre-mRNA may represent an important mechanism that controls the relative abundance of the full-length transcript and the level of protein, which in turn has a major impact on the MAPK/ERK signalling pathway. Moreover, our results suggest that ST5 is a negative regulator of both ERK1 and ERK2 activation.

The role of MAPK/ERK signalling has been implicated in many aspects of spermatogenesis including germ cell adhesion [49], [50]. Inhibition of upstream kinases (MEK1/2/5) of the ERK pathway was shown to delay germ cell loss induced by adjudin in the rat testis [50]. Similarly, hyperactivated ERK1/2 in the adult rat testis induced by an oral dose of the indenopyridine compound *l*-CDB-4022 was shown to significantly enhance germ cell loss [49]. Our results showed that spermatid differentiation arrest in the *Rbm5^sda/sda^* mice was accompanied by germ cell loss via sloughing. In line with studies in rat models [49], [50], we propose that hyperactivation of ERK1/2 contributed, at least in part, to germ cell loss in the *Rbm5^sda/sda^* mice.

The data contained within this manuscript strongly supports that the primary phenotype within the *Rbm5^sda/sda^* mice, male sterility, is due to RBM5 dysfunction within germ cells specifically. It should however be acknowledged that RBM5 is found within Sertoli cells (Figure 4C), thus Sertoli cell dysfunction may contribute to germ cell loss or abnormalities.

### RBM5 is likely to bind different target mRNAs in different tissues

In human cell lines, RBM5 has been previously shown to interact with a number of spliceosomal proteins [13], [15]. In round spermatids, we identified many hnRNPs, SR proteins and other splicing-related factors as RBM5 putative interacting partners. We confirmed that RBM5 directly interacted with SFPQ and hnRNP A2/B1. Subsequently, we identified 11 RBM5 putative target mRNAs within round spermatids. Interestingly, the four previously identified RBM5 targets in HeLa cells, *CASP2* [10], *FAS* and *c-FLIP* [13], and in B-lymphocytes, *AID* [14] were not detected in our RBM5 pull down, suggesting they are not RBM5 targets in round spermatids, and that RBM5 may interact with some of its target mRNAs in a tissue-specific manner.

Since it was identified over a decade ago [53], RBM5 has been proposed to play a key role in cell cycle and apoptosis regulation [54]–[58]. The data contained herein, supports such a role in apoptosis (as evidenced by activated Caspase staining), but also in the differentiation of male germ cells. The presentation of a mouse line with defective RBM5 with male sterility in isolation could be considered somewhat surprising given *Rbm5*'s wide expression profile and data showing an association between RBM5 and human lung cancer. In humans, RBM5 is one of 35 genes located within a 370 kb region on chromosome 3p21.3, which is frequently deleted in lung cancer [59]. RBM5 expression is down-regulated in primary lung cancer tissues [60] and has been linked to poor prognosis [20]. Together with several lines of *in vitro* evidence on its capacity to control cell cycle progression and apoptosis, RBM5 has been proposed as a putative tumour suppressor. Within our model of RBM5 dysfunction we observed no evidence of tumours or other histological abnormalities at 9 month-of-age under standard housing conditions. The possibility remains, however, that the *Rbm5^sda/sda^* allele retains some functional activity thus avoiding developmental defects, or that mice may ultimately develop tumours with advancing age.

In summary, our findings define RBM5 as a critical splicing regulator in round spermatids and demonstrate that the precise control of alternative splicing is crucial for spermiogenesis. Our data also suggests that the second RRM of RBM5 is pivotal for appropriate pre-mRNA splicing. Equally importantly, we have defined the physiological role for RBM5 in male fertility and revealed a pathway of potential male contraceptive targets, of diagnostic and/or therapeutic significance for the large number of men who fail to produce sperm.

## Materials and Methods

### Mouse strains

Animal experiments were approved by the Australian National University and the Monash University Animal Ethics committees. ENU mutagenesis was performed as previously described [17]–[19]. Comprehensive full body histology was performed at the University of Melbourne node of the Australian Phenomics Network (APN). The *Rbm5* knockout line was generated using an *Rbm5* gene-trap 129Sv ES cell line obtained from the Mammalian Functional Genomics Centre (Canada). The trapped ES clone was injected into C57BL/6J blastocyst to generate chimeric mice at the Monash node of the APN. The line was backcrossed to C57BL/6J for 10 generations. Genotyping of the *Rbm5^sda^* and knockout lines was performed using the Amplifluor SNP detection system as previously described [18], 19 and a standard PCR method, respectively. Primers are shown in (Supplementary Table S3). Comprehensive full body histology was performed at the University of Melbourne node of the APN.

### Antibodies

A synthetic peptide (CRERERRNSDRSEDG) encoding amino acid positions 62–75 of the mouse RBM5 protein was used to generate a monoclonal antibody at the Monash Antibody Technologies Facility as described previously [18]. Clones were negatively selected against using peptides corresponding to the equivalent regions of the closely related paralogous RBM6 and RBM10. The antibody used in our studies is called RBM5-clone A9. The antibody binding specificity was determined by pre-absorption of the antiserum using a 50-fold molar excess of the peptide. Antibodies purchased from commercial sources include: hnRNPA2B1 (Abcam, ab6102), SFPQ (Abcam, ab38148), ST5 (Abcam, ab64897), ERK1/2 (Sigma, #5670), p-ERK1/2 (Cell Signalling Technology, #4370), and HPRT (Abcam, ab10479). Western blotting, immunohistochemistry and immunofluorescence were performed as previously described [18], [19]. Germ cell apoptosis was assessed by immunostaining for cleaved Caspase 3 and cleaved Caspase 9 (Cell Signalling, #9664 and #9509). Quantitative analysis was performed by counting numbers of Caspase-positive cells in 250 tubules per testis (*n* = 3 mice per group).

### qRT-PCR

Total RNAs were extracted from C57BL/6JxCBA mice (*n* = 3 per tissue and per age group) using Trizol reagent (Invitrogen) and converted into cDNA using SuperScriptIII reverse transcriptase (Invitrogen). Predesigned TaqMan assays (Life technologies) were used to detect *Rbm5* expression (Mm00455721_m1). For the testis age survey, data were normalised to *Ppia* (Mm02342429_g1). For the different tissues expression pattern, data were normalised to *Hprt* (Mm00446968_m1). Testis expression was set to 100%. TaqMan assays used to verify RNA pull down experiments were *St5* (Mm00551615_m1), *Asb1* (Mm04206231) and *Pla2g10* (Mm01344436_g1).

### Protein pull down and mass spectrometry

Round spermatids from *Rbm5^WT/WT^* mice (*n* = 3) were purified using the Staput method as previously described [19]. Protein pull down was performed using 5 µg of the RBM5 mouse monoclonal antibody or mouse IgG1. Mass spectrometry analysis was performed at the Australian Proteomics Analysis Facility. Proteins with Mascot search score of at least 40 and more than 2 unique peptides matched were considered as putative RBM5 binding partners.

### Identification of RBM5 target mRNAs

RNA pull down using the RBM5 antibody and mouse IgG was performed using purified round spermatids as previously described [61]. Microarray was performed using Illumina mouse WG6v2 array at the Australian Genome Research Facility (AGRF). We conducted 3 independent pull down sets (from 3 biological replicates) and the level of each target was averaged. Enrichment scores (fold enrichment) were defined by the averaged level of each target versus their level of expression in the total round spermatid extracts (input material). Targets with ≥2-fold enrichment were defined as RBM5 targets. qRT-PCR was employed to determine the levels of *St5*, *Asb1* and *Pla2g10*, relative to *Ppia*, in the remaining RBM5 and IgG pulled down samples to verify the specificity of RNA pull down.

### Determination of splicing defects

RT-PCR analysis was performed using total RNAs from purified round spermatids of the *Rbm5^WT/WT^* and *Rbm5^sda/sda^* mice. cDNA conversion was performed using oligo dT as described above. 200 ng of cDNA was used as template for the subsequent PCR amplification step (Supplemental Table S3). *St5*, *Asb1* and *Pla2g10* alternatively spliced transcripts were further confirmed by sequencing.

### Hormone analysis

FSH and LH serum concentrations were measured as described previously [62]. Testosterone concentrations were measured using the Active Testosterone RIA DSL-4000 (Diagnostic Systems Laboratories, Webster TX).

### Microarray analyses

Round spermatids were purified from day 28 *Rbm5^sda/sda^* and *Rbm5^WT/WT^* mice (*n* = 3 per group) as described above. Microarray and bioinformatics analysis was performed at the AGRF using Illumina mouse WG6v2 arrays. Probe sets showing a ≥2-fold change were defined as differentially expressed genes and submitted to the DAVID Functional Clustering algorithm [26], [27] (http://david.abcc.ncifcrf.gov) in order to elucidate which biological functions were significantly enriched in the differentially expressed genes. The top most significant and relevant functional clusters/functions were noted, along with enrichment scores and p values.

## Supporting Information

Figure S1 Full immunoblotting images of RBM5 co-immunoprecipitation. RBM5-PD: RBM5 pulled down samples; IgG-PD: IgG pulled down samples; IgG was loaded in order to locate heavy and light chains. (TIF)

Table S1 Differentially expressed genes in *Rbm5^sda/sda^* round spermatids as determined by microarray analysis. (DOCX)

Table S2 RBM5 interacting proteins in round spermatids identified using immunoprecipitation and mass spectrometry analysis. (DOC)

Table S3 Primers used for genotyping and RT-PCR analysis. (DOCX)

## References

[1] McLachlan RI, O'Bryan MK (2010) Clinical Review#: State of the art for genetic testing of infertile men. J Clin Endocrinol Metab 95: 1013–1024. 20089613 10.1210/jc.2009-1925

[2] McLachlan RI, Rajpert-De Meyts E, Hoei-Hansen CE, de Kretser DM, Skakkebaek NE (2007) Histological evaluation of the human testis–approaches to optimizing the clinical value of the assessment: mini review. Hum Reprod 22: 2–16. 16887924 10.1093/humrep/del279

[3] Eddy EM (1998) Regulation of gene expression during spermatogenesis. Semin Cell Dev Biol 9: 451–457. 9813192 10.1006/scdb.1998.0201

[4] Lunde BM, Moore C, Varani G (2007) RNA-binding proteins: modular design for efficient function. Nat Rev Mol Cell Biol 8: 479–490. 17473849 10.1038/nrm2178 PMC5507177

[5] Pan Q, Shai O, Lee LJ, Frey BJ, Blencowe BJ (2008) Deep surveying of alternative splicing complexity in the human transcriptome by high-throughput sequencing. Nat Genet 40: 1413–1415. 18978789 10.1038/ng.259

[6] Lewis BP, Green RE, Brenner SE (2003) Evidence for the widespread coupling of alternative splicing and nonsense-mediated mRNA decay in humans. Proc Natl Acad Sci U S A 100: 189–192. 12502788 10.1073/pnas.0136770100 PMC140922

[7] Akaike Y, Kurokawa K, Kajita K, Kuwano Y, Masuda K, et al (2011) Skipping of an alternative intron in the srsf1 3′ untranslated region increases transcript stability. J Med Invest 58: 180–187. 21921418 10.2152/jmi.58.180

[8] Curnow KM, Pascoe L, Davies E, White PC, Corvol P, et al (1995) Alternatively spliced human type 1 angiotensin II receptor mRNAs are translated at different efficiencies and encode two receptor isoforms. Mol Endocrinol 9: 1250–1262. 7491117 10.1210/mend.9.9.7491117

[9] Caceres JF, Kornblihtt AR (2002) Alternative splicing: multiple control mechanisms and involvement in human disease. Trends Genet 18: 186–193. 11932019 10.1016/s0168-9525(01)02626-9

[10] Fushimi K, Ray P, Kar A, Wang L, Sutherland LC, et al (2008) Up-regulation of the proapoptotic caspase 2 splicing isoform by a candidate tumor suppressor, RBM5. Proc Natl Acad Sci U S A 105: 15708–15713. 18840686 10.1073/pnas.0805569105 PMC2572934

[11] Song Z, Wu P, Ji P, Zhang J, Gong Q, et al (2012) Solution Structure of the Second RRM Domain of RBM5 and Its Unusual Binding Characters for Different RNA Targets. Biochemistry 51: 6667–78. 22839758 10.1021/bi300539t

[12] Behzadnia N, Golas MM, Hartmuth K, Sander B, Kastner B, et al (2007) Composition and three-dimensional EM structure of double affinity-purified, human prespliceosomal A complexes. EMBO J 26: 1737–1748. 17332742 10.1038/sj.emboj.7601631 PMC1829389

[13] Bonnal S, Martinez C, Forch P, Bachi A, Wilm M, et al (2008) RBM5/Luca-15/H37 regulates Fas alternative splice site pairing after exon definition. Mol Cell 32: 81–95. 18851835 10.1016/j.molcel.2008.08.008

[14] Jin W, Niu Z, Xu D, Li X (2012) RBM5 promotes exon 4 skipping of AID pre-mRNA by competing with the binding of U2AF65 to the polypyrimidine tract. FEBS Lett 586: 3852–3857. 23017209 10.1016/j.febslet.2012.09.006

[15] Niu Z, Jin W, Zhang L, Li X (2012) Tumor suppressor RBM5 directly interacts with the DExD/H-box protein DHX15 and stimulates its helicase activity. FEBS Lett 586: 977–983. 22569250 10.1016/j.febslet.2012.02.052

[16] Sugliani M, Brambilla V, Clerkx EJ, Koornneef M, Soppe WJ (2010) The conserved splicing factor SUA controls alternative splicing of the developmental regulator ABI3 in Arabidopsis. Plant Cell 22: 1936–1946. 20525852 10.1105/tpc.110.074674 PMC2910958

[17] Jamsai D, O'Bryan MK (2010) Genome-wide ENU mutagenesis for the discovery of novel male fertility regulators. Syst Biol Reprod Med 56: 246–259. 20536324 10.3109/19396361003706424

[18] Lo JC, Jamsai D, O'Connor AE, Borg C, Clark BJ, et al (2012) RAB-Like 2 Has an Essential Role in Male Fertility, Sperm Intra-Flagellar Transport, and Tail Assembly. PLoS Genet 8: e1002969. 23055941 10.1371/journal.pgen.1002969 PMC3464206

[19] O'Donnell L, Rhodes D, Smith SJ, Merriner DJ, Clark BJ, et al (2012) An Essential Role for Katanin p80 and Microtubule Severing in Male Gamete Production. PLoS Genet 8: e1002698. 22654669 10.1371/journal.pgen.1002698 PMC3359970

[20] Oh JJ, Taschereau EO, Koegel AK, Ginther CL, Rotow JK, et al (2010) RBM5/H37 tumor suppressor, located at the lung cancer hot spot 3p21.3, alters expression of genes involved in metastasis. Lung Cancer 70: 253–262. 20338664 10.1016/j.lungcan.2010.02.012

[21] Sutherland LC, Wang K, Robinson AG (2010) RBM5 as a putative tumor suppressor gene for lung cancer. J Thorac Oncol 5: 294–298. 20186023 10.1097/JTO.0b013e3181c6e330

[22] Holdcraft RW, Braun RE (2004) Androgen receptor function is required in Sertoli cells for the terminal differentiation of haploid spermatids. Development 131: 459–467. 14701682 10.1242/dev.00957

[23] Hermo L, Pelletier RM, Cyr DG, Smith CE (2010) Surfing the wave, cycle, life history, and genes/proteins expressed by testicular germ cells. Part 3: developmental changes in spermatid flagellum and cytoplasmic droplet and interaction of sperm with the zona pellucida and egg plasma membrane. Microsc Res Tech 73: 320–363. 19941287 10.1002/jemt.20784

[24] Hermo L, Pelletier RM, Cyr DG, Smith CE (2010) Surfing the wave, cycle, life history, and genes/proteins expressed by testicular germ cells. Part 2: changes in spermatid organelles associated with development of spermatozoa. Microsc Res Tech 73: 279–319. 19941292 10.1002/jemt.20787

[25] Oliva R (2006) Protamines and male infertility. Hum Reprod Update 12: 417–435. 16581810 10.1093/humupd/dml009

[26] Huang da W, Sherman BT, Tan Q, Kir J, Liu D, et al (2007) DAVID Bioinformatics Resources: expanded annotation database and novel algorithms to better extract biology from large gene lists. Nucleic Acids Res 35: W169–175. 17576678 10.1093/nar/gkm415 PMC1933169

[27] Huang da W, Sherman BT, Lempicki RA (2009) Systematic and integrative analysis of large gene lists using DAVID bioinformatics resources. Nat Protoc 4: 44–57. 19131956 10.1038/nprot.2008.211

[28] Sun X, Kovacs T, Hu YJ, Yang WX (2011) The role of actin and myosin during spermatogenesis. Mol Biol Rep 38: 3993–4001. 21107714 10.1007/s11033-010-0517-0

[29] Kierszenbaum AL, Rivkin E, Tres LL (2003) Acroplaxome, an F-actin-keratin-containing plate, anchors the acrosome to the nucleus during shaping of the spermatid head. Mol Biol Cell 14: 4628–4640. 14551252 10.1091/mbc.E03-04-0226 PMC266778

[30] Kierszenbaum AL, Tres LL (2004) The acrosome-acroplaxome-manchette complex and the shaping of the spermatid head. Arch Histol Cytol 67: 271–284. 15700535 10.1679/aohc.67.271

[31] Kierszenbaum AL (2002) Intramanchette transport (IMT): managing the making of the spermatid head, centrosome, and tail. Mol Reprod Dev 63: 1–4. 12211054 10.1002/mrd.10179

[32] O'Brien DA, Gabel CA, Welch JE, Eddy EM (1991) Mannose 6-phosphate receptors: potential mediators of germ cell-Sertoli cell interactions. Ann N Y Acad Sci 637: 327–339. 1664679 10.1111/j.1749-6632.1991.tb27320.x

[33] Kile BT, Metcalf D, Mifsud S, DiRago L, Nicola NA, et al (2001) Functional analysis of Asb-1 using genetic modification in mice. Mol Cell Biol 21: 6189–6197. 11509662 10.1128/MCB.21.18.6189-6197.2001 PMC87336

[34] Escoffier J, Pierre VJ, Jemel I, Munch L, Boudhraa Z, et al (2011) Group X secreted phospholipase A(2) specifically decreases sperm motility in mice. J Cell Physiol 226: 2601–2609. 21792918 10.1002/jcp.22606

[35] Escoffier J, Jemel I, Tanemoto A, Taketomi Y, Payre C, et al (2010) Group X phospholipase A2 is released during sperm acrosome reaction and controls fertility outcome in mice. J Clin Invest 120: 1415–1428. 20424324 10.1172/JCI40494 PMC2860919

[36] Saade M, Irla M, Govin J, Victorero G, Samson M, et al (2007) Dynamic distribution of Spatial during mouse spermatogenesis and its interaction with the kinesin KIF17b. Exp Cell Res 313: 614–626. 17196196 10.1016/j.yexcr.2006.11.011

[37] Dishinger JF, Kee HL, Jenkins PM, Fan S, Hurd TW, et al (2010) Ciliary entry of the kinesin-2 motor KIF17 is regulated by importin-beta2 and RanGTP. Nat Cell Biol 12: 703–710. 20526328 10.1038/ncb2073 PMC2896429

[38] Bauer H, Willert J, Koschorz B, Herrmann BG (2005) The t complex-encoded GTPase-activating protein Tagap1 acts as a transmission ratio distorter in mice. Nat Genet 37: 969–973. 16116428 10.1038/ng1617

[39] Elliott DJ, Grellscheid SN (2006) Alternative RNA splicing regulation in the testis. Reproduction 132: 811–819. 17127741 10.1530/REP-06-0147

[40] Imataka H, Gradi A, Sonenberg N (1998) A newly identified N-terminal amino acid sequence of human eIF4G binds poly(A)-binding protein and functions in poly(A)-dependent translation. EMBO J 17: 7480–7489. 9857202 10.1093/emboj/17.24.7480 PMC1171091

[41] Chi MN, Auriol J, Jegou B, Kontoyiannis DL, Turner JM, et al (2011) The RNA-binding protein ELAVL1/HuR is essential for mouse spermatogenesis, acting both at meiotic and postmeiotic stages. Mol Biol Cell 22: 2875–2885. 21737689 10.1091/mbc.E11-03-0212 PMC3154883

[42] Liu N, Han H, Lasko P (2009) Vasa promotes Drosophila germline stem cell differentiation by activating mei-P26 translation by directly interacting with a (U)-rich motif in its 3′ UTR. Genes Dev 23: 2742–2752. 19952109 10.1101/gad.1820709 PMC2788330

[43] Salton M, Elkon R, Borodina T, Davydov A, Yaspo ML, et al (2011) Matrin 3 binds and stabilizes mRNA. PLoS One 6: e23882. 21858232 10.1371/journal.pone.0023882 PMC3157474

[44] Majidi M, Gutkind JS, Lichy JH (2000) Deletion of the COOH terminus converts the ST5 p70 protein from an inhibitor of RAS signaling to an activator with transforming activity in NIH-3T3 cells. J Biol Chem 275: 6560–6565. 10692462 10.1074/jbc.275.9.6560

[45] Majidi M, Hubbs AE, Lichy JH (1998) Activation of extracellular signal-regulated kinase 2 by a novel Abl-binding protein, ST5. J Biol Chem 273: 16608–16614. 9632734 10.1074/jbc.273.26.16608

[46] Gupta RK, Gao N, Gorski RK, White P, Hardy OT, et al (2007) Expansion of adult beta-cell mass in response to increased metabolic demand is dependent on HNF-4alpha. Genes Dev 21: 756–769. 17403778 10.1101/gad.1535507 PMC1838528

[47] Kile BT, Alexander WS (2001) The suppressors of cytokine signalling (SOCS). Cell Mol Life Sci 58: 1627–1635. 11706989 10.1007/PL00000801 PMC11337286

[48] Li MW, Mruk DD, Cheng CY (2009) Mitogen-activated protein kinases in male reproductive function. Trends Mol Med 15: 159–168. 19303360 10.1016/j.molmed.2009.02.002 PMC2804913

[49] Koduri S, Hild SA, Pessaint L, Reel JR, Attardi BJ (2008) Mechanism of action of l-CDB-4022, a potential nonhormonal male contraceptive, in the seminiferous epithelium of the rat testis. Endocrinology 149: 1850–1860. 18174280 10.1210/en.2007-1332 PMC2276710

[50] Xia W, Cheng CY (2005) TGF-beta3 regulates anchoring junction dynamics in the seminiferous epithelium of the rat testis via the Ras/ERK signaling pathway: An in vivo study. Dev Biol 280: 321–343. 15882576 10.1016/j.ydbio.2004.12.036

[51] Maris C, Dominguez C, Allain FH (2005) The RNA recognition motif, a plastic RNA-binding platform to regulate post-transcriptional gene expression. FEBS J 272: 2118–2131. 15853797 10.1111/j.1742-4658.2005.04653.x

[52] Kerr JB (1991) Ultrastructure of the seminiferous epithelium and intertubular tissue of the human testis. J Electron Microsc Tech 19: 215–240. 1748903 10.1002/jemt.1060190208

[53] Oh JJ, Grosshans DR, Wong SG, Slamon DJ (1999) Identification of differentially expressed genes associated with HER-2/neu overexpression in human breast cancer cells. Nucleic Acids Res 27: 4008–4017. 10497265 10.1093/nar/27.20.4008 PMC148668

[54] Mourtada-Maarabouni M, Sutherland LC, Williams GT (2002) Candidate tumour suppressor LUCA-15 can regulate multiple apoptotic pathways. Apoptosis 7: 421–432. 12207175 10.1023/a:1020083008017

[55] Mourtada-Maarabouni M, Sutherland LC, Meredith JM, Williams GT (2003) Simultaneous acceleration of the cell cycle and suppression of apoptosis by splice variant delta-6 of the candidate tumour suppressor LUCA-15/RBM5. Genes Cells 8: 109–119. 12581154 10.1046/j.1365-2443.2003.00619.x

[56] Mourtada-Maarabouni M, Keen J, Clark J, Cooper CS, Williams GT (2006) Candidate tumor suppressor LUCA-15/RBM5/H37 modulates expression of apoptosis and cell cycle genes. Exp Cell Res 312: 1745–1752. 16546166 10.1016/j.yexcr.2006.02.009

[57] Oh JJ, Razfar A, Delgado I, Reed RA, Malkina A, et al (2006) 3p21.3 tumor suppressor gene H37/Luca15/RBM5 inhibits growth of human lung cancer cells through cell cycle arrest and apoptosis. Cancer Res 66: 3419–3427. 16585163 10.1158/0008-5472.CAN-05-1667

[58] Rintala-Maki ND, Sutherland LC (2004) LUCA-15/RBM5, a putative tumour suppressor, enhances multiple receptor-initiated death signals. Apoptosis 9: 475–484. 15192330 10.1023/B:APPT.0000031455.79352.57

[59] Wei MH, Latif F, Bader S, Kashuba V, Chen JY, et al (1996) Construction of a 600-kilobase cosmid clone contig and generation of a transcriptional map surrounding the lung cancer tumor suppressor gene (TSG) locus on human chromosome 3p21.3: progress toward the isolation of a lung cancer TSG. Cancer Res 56: 1487–1492. 8603390

[60] Oh JJ, West AR, Fishbein MC, Slamon DJ (2002) A candidate tumor suppressor gene, H37, from the human lung cancer tumor suppressor locus 3p21.3. Cancer Res 62: 3207–3213. 12036935

[61] Monk AC, Siddall NA, Volk T, Fraser B, Quinn LM, et al (2010) HOW is required for stem cell maintenance in the Drosophila testis and for the onset of transit-amplifying divisions. Cell Stem Cell 6: 348–360. 20362539 10.1016/j.stem.2010.02.016

[62] Kennedy CL, O'Connor AE, Sanchez-Partida LG, Holland MK, Goodnow CC, et al (2005) A repository of ENU mutant mouse lines and their potential for male fertility research. Mol Hum Reprod 11: 871–880. 16421219 10.1093/molehr/gah251

[63] Bischoff FR, Krebber H, Kempf T, Hermes I, Ponstingl H (1995) Human RanGTPase-activating protein RanGAP1 is a homologue of yeast Rna1p involved in mRNA processing and transport. Proc Natl Acad Sci U S A 92: 1749–1753. 7878053 10.1073/pnas.92.5.1749 PMC42597

[64] Xu M, Luo W, Elzi DJ, Grandori C, Galloway DA (2008) NFX1 interacts with mSin3A/histone deacetylase to repress hTERT transcription in keratinocytes. Mol Cell Biol 28: 4819–4828. 18505829 10.1128/MCB.01969-07 PMC2493374

[65] Wong PY (1998) CFTR gene and male fertility. Mol Hum Reprod 4: 107–110. 9542966 10.1093/molehr/4.2.107

