# Supplementary figures and images for: RBM5 Is a Male Germ Cell Splicing Factor and Is Required for Spermatid Differentiation and Male Fertility
Source: PLoS Genet. 2013 Jul 25;9(7):e1003628. doi: 10.1371/journal.pgen.1003628 (PMC3723494; doi:10.1371/journal.pgen.1003628)

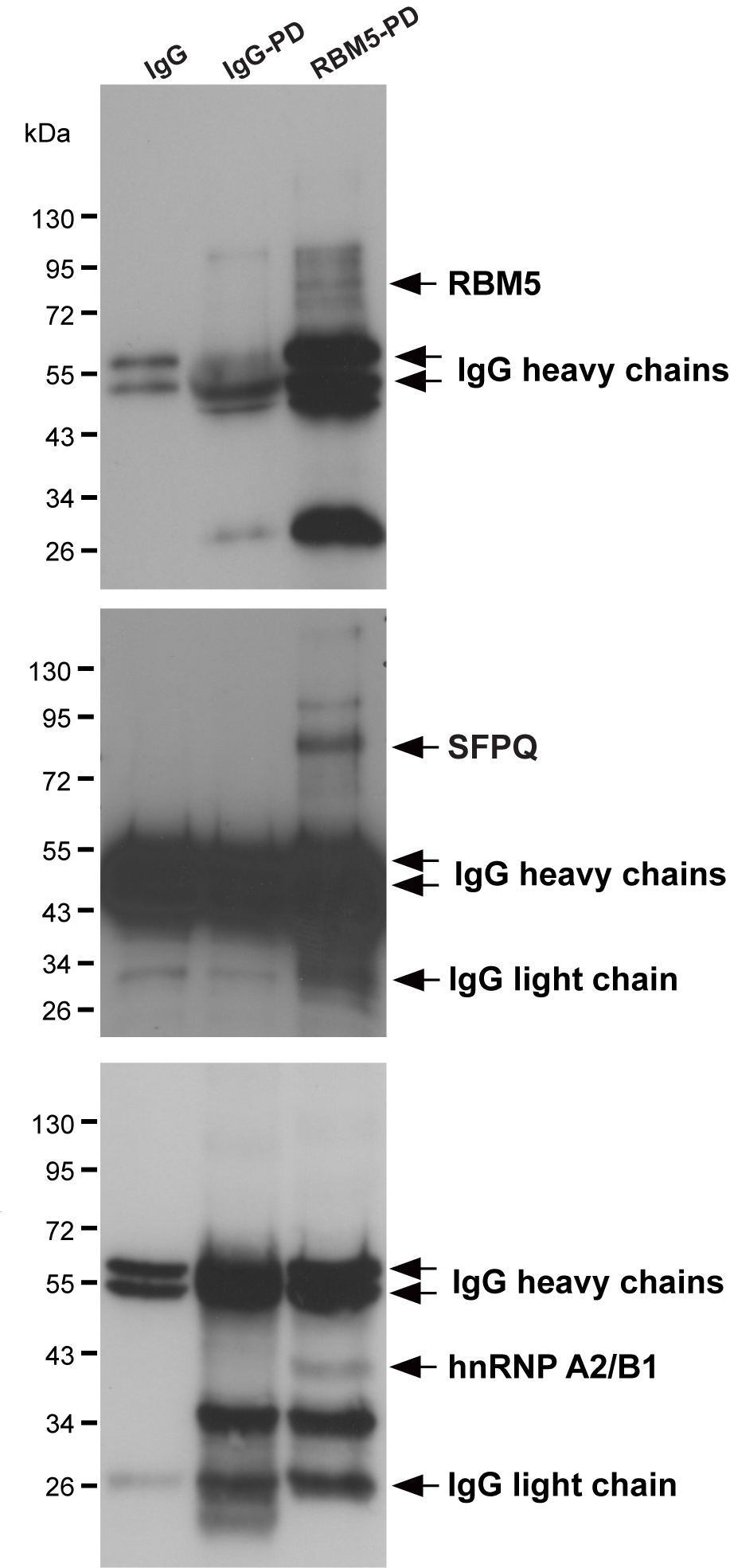

Supplement: Figure S1 — (TIF) [file pgen.1003628.s001.tif]
