# Supplementary material for: RBM5 Is a Male Germ Cell Splicing Factor and Is Required for Spermatid Differentiation and Male Fertility
Source: PLoS Genet. 2013 Jul 25;9(7):e1003628. doi: 10.1371/journal.pgen.1003628 (PMC3723494; doi:10.1371/journal.pgen.1003628)
Supplement: Table S1 — (DOCX) [file pgen.1003628.s002.docx]

**Supplementary Table S1: Differentially expressed genes in *Rbm5^sda/sda^* round spermatids as determined by microarray analysis.**

| **Down-regulated genes** | | | | |
| --- | --- | --- | --- | --- |
| **Probeset ID** | **Transcript** | **Gene Symbol** | **Accession** | **Fold-Change WT vs. Mutant** |
| 4040164 | ILMN_190617 | 1700104B16Rik |  | -14.91 |
| 2490332 | ILMN_222601 | BC061237 | NM_198677.1 | -14.04 |
| 6330075 | ILMN_320574 | Syce1 | XM_001479666.1 | -9.15 |
| 2360474 | ILMN_257853 | EG638695 | NM_001039250.1 | -7.42 |
| 2600204 | ILMN_215008 | Arl3 | NM_019718.2 | -7.23 |
| 6770224 | ILMN_258759 | LOC545952 | NM_001024727.1 | -6.31 |
| 5550288 | ILMN_215234 | Sh2d6 | XM_355785.4 | -5.86 |
| 2570487 | ILMN_209838 | H2-Ab1 | NM_207105.2 | -5.82 |
| 5960327 | ILMN_223726 | Cox7a2l | XM_123188.1 | -5.68 |
| 7100367 | ILMN_316241 | 1700124P09Rik | XM_001481019.1 | -5.56 |
| 240075 | ILMN_327750 | LOC100040863 | XR_032231.1 | -5.27 |
| 4070634 | ILMN_321503 | ENSMUSG00000044227 | XM_001480668.1 | -5.01 |
| 6370333 | ILMN_215234 | Sh2d6 | XM_355785.4 | -4.96 |
| 2000500 | ILMN_191853 | 4930432J01Rik |  | -4.68 |
| 7380079 | ILMN_215234 | Sh2d6 | XM_355785.4 | -4.66 |
| 7320056 | ILMN_221143 | Bat5 | NM_178592.3 | -4.56 |
| 2070519 | ILMN_201751 | Nnmt | AK006371 | -4.55 |
| 4120685 | ILMN_201687 | 2610028H07Rik | AK011590 | -4.48 |
| 4010424 | ILMN_192139 | 4930405J17Rik |  | -4.34 |
| 3850201 | ILMN_201911 | 4933439F18Rik | AK017120 | -4.33 |
| 780315 | ILMN_230698 | LOC641240 | XM_918601.3 | -4.28 |
| 5340328 | ILMN_187056 | Zfp330 | NM_145600.1 | -4.27 |
| 3780553 | ILMN_241004 | EG237009 | NM_207241.1 | -4.22 |
| 6560497 | ILMN_215936 | Nnmt | NM_010924.1 | -4.19 |
| 610762 | ILMN_221121 | Cops8 | NM_133805.3 | -4.17 |
| 5690343 | ILMN_212979 | Tbc1d2 | NM_198664.3 | -4.14 |
| 2450669 | ILMN_192161 | 1700040A22Rik |  | -4.06 |
| 3170739 | ILMN_229687 | Ccl27 | NM_001048179.1 | -3.93 |
| 6290112 | ILMN_225380 | EG545047 | NM_001034102.1 | -3.88 |
| 1820022 | ILMN_210775 | Csl | NM_027945.1 | -3.84 |
| 670360 | ILMN_220148 | Tubb6 | NM_026473.2 | -3.79 |
| 7210193 | ILMN_224562 | 4933413N12Rik | XR_001594.1 | -3.79 |
| 3370678 | ILMN_201553 | Ssty1 | NM_009220.1 | -3.62 |
| 6520546 | ILMN_318926 | LOC100042481 | XM_001477627.1 | -3.55 |
| 4250646 | ILMN_230701 | 3110007F17Rik | NM_028426.1 | -3.48 |
| 20706 | ILMN_210832 | Tmem144 | NM_027495.2 | -3.48 |
| 5700133 | ILMN_315555 | Ptar1 | XM_001477013.1 | -3.48 |
| 4760110 | ILMN_216700 | Itgb1bp1 | NM_008403.2 | -3.41 |
| 1570132 | ILMN_197962 | LOC239727 | XM_156408.2 | -3.38 |
| 5860139 | ILMN_223599 | Chchd8 | NM_183270.2 | -3.34 |
| 3420736 | ILMN_209733 | Mff | NM_029409.2 | -3.34 |
| 3940092 | ILMN_252173 | Pop4 | NM_025390.4 | -3.30 |
| 540259 | ILMN_197814 | LOC230253 | XM_124269.2 | -3.29 |
| 4280156 | ILMN_220472 | Thumpd1 | NM_145585.1 | -3.27 |
| 1570187 | ILMN_233236 | Ipmk | NM_027184.1 | -3.19 |
| 2940164 | ILMN_216653 | Rbm13 | NM_026453.1 | -3.18 |
| 5340358 | ILMN_216432 | Ccl27 | NM_011336.1 | -3.16 |
| 5390328 | ILMN_209495 | Dcn | NM_007833.4 | -3.15 |
| 3400615 | ILMN_209260 | Rps3 | NM_012052.1 | -3.12 |
| 6580112 | ILMN_188949 | Zfp393 | NM_029416.1 | -3.10 |
| 130347 | ILMN_225474 | Sly | NM_201530.1 | -3.10 |
| 4210170 | ILMN_200813 | LOC382213 | XM_356326.1 | -3.08 |
| 4250315 | ILMN_202704 | Mscp-pending | AK019700 | -3.05 |
| 2490538 | ILMN_233220 | B230311B06Rik | XM_001001884.2 | -3.05 |
| 1850189 | ILMN_189182 | 1500035N22Rik |  | -3.03 |
| 2350670 | ILMN_231978 | 2810032E02Rik | XM_894566.3 | -3.02 |
| 4900025 | ILMN_200373 | LOC386360 | XM_359184.1 | -3.00 |
| 4280577 | ILMN_235895 | EG546282 | NM_001081657.1 | -3.00 |
| 1500341 | ILMN_252784 | Thap4 | NM_025920.3 | -2.99 |
| 2570184 | ILMN_197620 | LOC268730 | XM_193754.2 | -2.97 |
| 2600270 | ILMN_239643 | 1700091H14Rik | XM_983279.2 | -2.97 |
| 3710242 | ILMN_210252 | Arpc3 | NM_019824.3 | -2.92 |
| 6840669 | ILMN_228895 | LOC665918 | XM_980351.1 | -2.92 |
| 1740112 | ILMN_245559 | Loxl1 | NM_010729.2 | -2.89 |
| 2190162 | ILMN_311715 | LOC100040020 | XM_001473955.1 | -2.88 |
| 5870762 | ILMN_200027 | LOC383125 | XM_356890.1 | -2.87 |
| 6420138 | ILMN_198034 | LOC332788 | XM_285750.2 | -2.86 |
| 1400373 | ILMN_321979 | Ube2dnl | NR_003645.1 | -2.83 |
| 7550593 | ILMN_254040 | 1700016D18Rik | XM_986799.1 | -2.83 |
| 6580575 | ILMN_225494 | 1700021F07Rik | XM_913802.2 | -2.79 |
| 6760341 | ILMN_247941 | LOC667034 | XM_992876.1 | -2.79 |
| 4730307 | ILMN_210775 | Csl | NM_027945.1 | -2.76 |
| 7510356 | ILMN_217996 | Insig2 | NM_133748.1 | -2.76 |
| 1440307 | ILMN_209838 | H2-Ab1 | NM_207105.2 | -2.74 |
| 4290458 | ILMN_207130 | F730003H07Rik | AK089303 | -2.73 |
| 1170671 | ILMN_229145 | Rbpms | NM_019733.2 | -2.73 |
| 7570382 | ILMN_222089 | Fth1 | NM_010239.1 | -2.72 |
| 2970709 | ILMN_187478 | Bcl11a | NM_016707 | -2.72 |
| 3130433 | ILMN_220647 | Ttc27 | NM_152817.3 | -2.72 |
| 6480484 | ILMN_221737 | Tgif2lx | NM_153109.1 | -2.71 |
| 1170687 | ILMN_221704 | 0610009B14Rik | XM_127006.1 | -2.69 |
| 6960044 | ILMN_196566 | Atpaf2 | NM_145427.1 | -2.67 |
| 4900021 | ILMN_207069 | 6430590A07Rik | AK078307 | -2.66 |
| 4050270 | ILMN_200358 | LOC386294 | XM_359162.1 | -2.65 |
| 6590168 | ILMN_225468 | LOC638024 | XM_913893.3 | -2.64 |
| 1940576 | ILMN_232052 | MGC118210 | NM_001025607.1 | -2.64 |
| 1850239 | ILMN_200560 | LOC384348 | XM_357593.1 | -2.63 |
| 6400711 | ILMN_240127 | Cypt2 | NM_173436.1 | -2.62 |
| 1190463 | ILMN_220476 | 1700011K15Rik | NM_029294.1 | -2.62 |
| 6940747 | ILMN_311087 | LOC100041980 | XM_001476551.1 | -2.62 |
| 6840112 | ILMN_194014 | Uap1 | NM_133806.4 | -2.62 |
| 2970750 | ILMN_223860 | Cpn1 | NM_030703 | -2.60 |
| 6450739 | ILMN_240151 | LOC434960 | NM_001025241.1 | -2.60 |
| 4730040 | ILMN_188042 | C230082I21Rik |  | -2.58 |
| 1580689 | ILMN_216043 | Anxa11 | NM_013469.1 | -2.57 |
| 2900278 | ILMN_186932 | 4930547E08Rik |  | -2.56 |
| 630100 | ILMN_209857 | Defb11 | NM_139221.1 | -2.56 |
| 3520500 | ILMN_209599 | 1700123O20Rik | NM_021437.1 | -2.56 |
| 2710632 | ILMN_243664 | Vps35 | NM_022997.4 | -2.54 |
| 130082 | ILMN_221037 | Nkx6-1 | NM_144955.2 | -2.54 |
| 4570577 | ILMN_250371 | LOC382133 | NM_207162.1 | -2.53 |
| 7050747 | ILMN_315559 | LOC100042290 | XR_033106.1 | -2.53 |
| 2370376 | ILMN_202050 | Ndel1 | AK011168 | -2.53 |
| 20368 | ILMN_211201 | 1700010D01Rik | NM_029590.2 | -2.53 |
| 3450368 | ILMN_252075 | LOC665746 | XM_979162.1 | -2.49 |
| 6960474 | ILMN_255921 | MGC107098 | NM_001017393.1 | -2.48 |
| 5390113 | ILMN_236251 | OTTMUSG00000016790 | NM_001025260.2 | -2.47 |
| 3890762 | ILMN_225115 | 2600011E07Rik | NM_028113.1 | -2.47 |
| 7150162 | ILMN_320101 | LOC100042550 | XM_001477781.1 | -2.47 |
| 1990546 | ILMN_235169 | Faim3 | NM_026976.2 | -2.46 |
| 5670341 | ILMN_196356 | EG330513 | NM_177890.3 | -2.46 |
| 3400703 | ILMN_217502 | Macrod1 | NM_134147.3 | -2.46 |
| 50133 | ILMN_324608 | 6530404N21Rik | XM_001481327.1 | -2.45 |
| 2190446 | ILMN_223711 | Pcdhb3 | NM_053128.2 | -2.45 |
| 3460113 | ILMN_316540 | LOC100042351 | XM_001477266.1 | -2.45 |
| 3800372 | ILMN_224024 | Alox5ap | NM_009663 | -2.45 |
| 5290360 | ILMN_209744 | Picalm | NM_146194 | -2.44 |
| 1030731 | ILMN_211018 | Mipep | NM_027436.2 | -2.43 |
| 3290187 | ILMN_211192 | Psmb5 | NM_011186.1 | -2.43 |
| 6520132 | ILMN_226343 | LOC665012 | XR_001681.1 | -2.42 |
| 4180470 | ILMN_199449 | LOC383483 | XM_357088.1 | -2.41 |
| 6060376 | ILMN_217436 | Orc6l | NM_019716.1 | -2.40 |
| 2480427 | ILMN_212979 | Tbc1d2 | NM_198664.3 | -2.39 |
| 5360349 | ILMN_186471 | Trim11 | NM_053168.1 | -2.39 |
| 5860435 | ILMN_209838 | H2-Ab1 | NM_207105.1 | -2.39 |
| 6280113 | ILMN_244045 | EG546250 | XM_486530.3 | -2.39 |
| 4250670 | ILMN_213138 | Cab39l | NM_026908.3 | -2.38 |
| 5860356 | ILMN_260376 | Rps6ka1 | NM_009097.1 | -2.38 |
| 6860672 | ILMN_245539 | LOC666096 | XM_981599.1 | -2.37 |
| 4010288 | ILMN_202403 | 4930599N23Rik | AK016417 | -2.36 |
| 1030594 | ILMN_329237 | LOC670593 | XM_001475988.1 | -2.35 |
| 6550382 | ILMN_253733 | Tmem55b | NM_001033271.3 | -2.35 |
| 5050445 | ILMN_237369 | 2610002J02Rik | NM_001033134.2 | -2.34 |
| 5340747 | ILMN_232177 | Gm525 | NM_001033266.2 | -2.34 |
| 940356 | ILMN_218011 | Tfb1m | NM_146074.1 | -2.33 |
| 2100386 | ILMN_218080 | Ndufb10 | XM_128594.4 | -2.33 |
| 520176 | ILMN_215222 | Pkd1 | NM_013630.2 | -2.33 |
| 2190392 | ILMN_309912 | LOC100041256 | XM_001475559.1 | -2.32 |
| 1170100 | ILMN_310186 | LOC100039693 | XM_001473202.1 | -2.32 |
| 670369 | ILMN_257790 | LOC631784 | NM_001039240.1 | -2.31 |
| 6180008 | ILMN_201010 | LOC385542 | XM_358275.1 | -2.30 |
| 1780170 | ILMN_216745 | Plekha4 | NM_148927.1 | -2.30 |
| 6200494 | ILMN_211934 | Sgca | NM_009161.3 | -2.30 |
| 6350379 | ILMN_248255 | Ssty2 | NM_023546.2 | -2.28 |
| 4850079 | ILMN_260615 | Kif23 | NM_024245.3 | -2.28 |
| 2650435 | ILMN_218794 | Csnk1d | NM_027874.2 | -2.28 |
| 5700088 | ILMN_188949 | Klf17 | NM_029416.2 | -2.27 |
| 4260220 | ILMN_231908 | E230008N13Rik | XM_975070.1 | -2.25 |
| 5690709 | ILMN_242965 | 4930524E20Rik | NM_001081661.1 | -2.24 |
| 6900544 | ILMN_317334 | LOC100039120 | XM_001472427.1 | -2.24 |
| 6480196 | ILMN_251757 | Dusp21 | NM_028568.1 | -2.24 |
| 4890435 | ILMN_312290 | LOC100045423 | XM_001472536.1 | -2.22 |
| 4120647 | ILMN_214731 | Fscn3 | NM_019569.2 | -2.22 |
| 5910164 | ILMN_250777 | Emilin2 | NM_145158.2 | -2.21 |
| 2690615 | ILMN_201007 | LOC382297 | XM_356416.1 | -2.21 |
| 6330707 | ILMN_206077 | E130009M23Rik | AK053322 | -2.21 |
| 6550537 | ILMN_217863 | Ribc1 | NM_025660.1 | -2.21 |
| 5490301 | ILMN_195734 | Tnk1 | NM_031880.2 | -2.20 |
| 6770463 | ILMN_256260 | 4930430D24Rik | NM_001034856.2 | -2.19 |
| 1780156 | ILMN_215570 | Maged1 | NM_019791.2 | -2.18 |
| 6840255 | ILMN_252375 | LOC631002 | NM_001081565.1 | -2.18 |
| 7330433 | ILMN_224730 | Rbm31y | XM_486938.3 | -2.18 |
| 4220639 | ILMN_310507 | LOC546015 | XR_034790.1 | -2.17 |
| 3390762 | ILMN_316185 | EG434758 | XM_001474585.1 | -2.17 |
| 1010703 | ILMN_210106 | Atp6v0e2 | NM_133764.2 | -2.16 |
| 5310075 | ILMN_202373 | Mscp-pending | AK015790 | -2.16 |
| 3420521 | ILMN_210079 | 6330503K22Rik | NM_182995.1 | -2.16 |
| 2030327 | ILMN_210813 | Adam6 | NM_174885.3 | -2.16 |
| 7210521 | ILMN_211345 | Pja1 | NM_008853.2 | -2.16 |
| 7610730 | ILMN_220404 | Dctn6 | NM_011722.2 | -2.16 |
| 3520192 | ILMN_220427 | Srpr | NM_026130.1 | -2.15 |
| 4880273 | ILMN_190589 | 4930442E04Rik |  | -2.15 |
| 5670670 | ILMN_203675 | 9530039I19Rik | AK035426 | -2.15 |
| 6020324 | ILMN_255958 | Fxr2 | NM_011814.2 | -2.15 |
| 2340382 | ILMN_210264 | Htatip2 | NM_016865.2 | -2.14 |
| 130039 | ILMN_212810 | Emd | NM_007927.2 | -2.14 |
| 5860379 | ILMN_196512 | 4933401B06Rik | XM_136018.3 | -2.14 |
| 770520 | ILMN_225536 | 4930428D18Rik | NM_001033799.1 | -2.13 |
| 2100195 | ILMN_210652 | 1700010M22Rik | NM_025490.1 | -2.13 |
| 5220131 | ILMN_309882 | 1700021K14Rik | XM_001475204.1 | -2.12 |
| 2650274 | ILMN_217620 | Car15 | NM_030558.2 | -2.12 |
| 3400154 | ILMN_314963 | 5830454E08Rik | XM_001473897.1 | -2.11 |
| 990427 | ILMN_213214 | 1700008E09Rik | NM_027055 | -2.11 |
| 5810246 | ILMN_258500 | Dnajc2 | NM_009584.3 | -2.11 |
| 5360474 | ILMN_207467 | Pi4k2b | NM_028744.2 | -2.11 |
| 3830739 | ILMN_225508 | Nupl2 | NM_153092.3 | -2.10 |
| 510343 | ILMN_258437 | Osbpl7 | NM_001081434.1 | -2.10 |
| 7320343 | ILMN_215217 | Wdr46 | NM_020603.2 | -2.09 |
| 3130709 | ILMN_232177 | Gm525 | NM_001033266.2 | -2.09 |
| 5340605 | ILMN_239121 | 8030474K03Rik | XM_001000772.2 | -2.08 |
| 1240653 | ILMN_206790 | Srr | AK080830 | -2.08 |
| 1580386 | ILMN_228617 | D830030K20Rik | NM_177135.2 | -2.08 |
| 4590114 | ILMN_193137 | scl0002791.1_134 |  | -2.08 |
| 4670468 | ILMN_248374 | Il17d | NM_145837.1 | -2.07 |
| 6520202 | ILMN_231407 | OTTMUSG00000017827 | NM_001081476.1 | -2.07 |
| 1580280 | ILMN_222089 | Fth1 | NM_010239.1 | -2.07 |
| 3390242 | ILMN_201446 | LOC382165 | XM_356265.1 | -2.07 |
| 6180379 | ILMN_232926 | LOC545013 | NM_001025085.1 | -2.07 |
| 3460070 | ILMN_216887 | Riok1 | NM_024242.2 | -2.07 |
| 2190239 | ILMN_209831 | Morf4l2 | NM_019768.2 | -2.06 |
| 2630707 | ILMN_188949 | Klf17 | NM_029416.2 | -2.06 |
| 6100059 | ILMN_220786 | Rhox3a | NM_194063.2 | -2.06 |
| 1710427 | ILMN_225115 | Fam123a | NM_028113.2 | -2.06 |
| 4390131 | ILMN_222214 | Tmem66 | NM_026432.2 | -2.05 |
| 2690288 | ILMN_215537 | Spnb1 | NM_013675 | -2.05 |
| 6980110 | ILMN_331077 | 1700049L16Rik | NR_003644.1 | -2.05 |
| 510474 | ILMN_222956 | Lgals3 | NM_010705.2 | -2.05 |
| 1070482 | ILMN_213874 | Mrpl48 | NM_026971.2 | -2.04 |
| 6980670 | ILMN_250002 | 4930487D11Rik | XM_979477.1 | -2.04 |
| 5130035 | ILMN_184803 | 1700084M14Rik |  | -2.03 |
| 6520291 | ILMN_253049 | Spaca4 | NM_027055.1 | -2.03 |
| 6180164 | ILMN_189815 | Nrg3 | NM_008734 | -2.03 |
| 3990435 | ILMN_212418 | Mgmt | NM_008598.1 | -2.02 |
| 4900091 | ILMN_215378 | 4930519G04Rik | NM_026263.2 | -2.02 |
| 7610390 | ILMN_192626 | Trpc2 | NM_011644.2 | -2.02 |
| 5490112 | ILMN_230898 | LOC670731 | XM_982997.1 | -2.02 |
| 5890440 | ILMN_235218 | Gm906 | NM_001033438.1 | -2.01 |
| 4150719 | ILMN_201519 | Speer4a | NM_029376.2 | -2.01 |
| 6840349 | ILMN_221409 | Pcdhgb2 | NM_033575.3 | -2.01 |
| 5130121 | ILMN_323018 | Tsga8 | XR_035356.1 | -2.00 |
| **Up-regulated genes** | | | | |
| **Probeset ID** | **Transcript** | **Gene Symbol** | **Accession** | **Fold-Change WT vs. Mutant** |
| 5700612 | ILMN_227876 | Ttc15 | NM_178811.3 | 2.00 |
| 4570739 | ILMN_213689 | Brf1 | NM_028193.3 | 2.00 |
| 5560605 | ILMN_203260 | Kif7 | AK034273 | 2.02 |
| 5220072 | ILMN_201922 | 4933432P15Rik | AK017029 | 2.02 |
| 2510041 | ILMN_318833 | Ndst2 | NM_010811.2 | 2.02 |
| 6900598 | ILMN_201766 | 2310007G05Rik | AK009204 | 2.02 |
| 5820255 | ILMN_232863 | Spatc1 | NM_028852.1 | 2.03 |
| 2650397 | ILMN_215505 | D4Bwg1540e | NM_026257.1 | 2.03 |
| 7330093 | ILMN_259837 | Ihpk2 | NM_029634.1 | 2.04 |
| 4610468 | ILMN_210012 | Cyb5r4 | NM_024195.1 | 2.04 |
| 3440343 | ILMN_214505 | Cap1 | NM_007598.2 | 2.04 |
| 2650630 | ILMN_196989 | LOC238333 | XM_138106.2 | 2.04 |
| 60398 | ILMN_225024 | Rffl | NM_001007465.1 | 2.05 |
| 5360082 | ILMN_188054 | Msr1 | M59445 | 2.05 |
| 540594 | ILMN_323432 | 1700120K04Rik | XR_035446.1 | 2.05 |
| 6040609 | ILMN_242220 | BB014433 | NM_001007591.1 | 2.05 |
| 2000398 | ILMN_216083 | Hba-a1 | NM_008218.2 | 2.06 |
| 780017 | ILMN_221060 | Spata3 | NM_027029.1 | 2.06 |
| 4070750 | ILMN_219287 | Spata3 | NM_027300.2 | 2.06 |
| 2940681 | ILMN_211079 | Gata2 | NM_008090.4 | 2.06 |
| 1450162 | ILMN_317709 | 1700042G07Rik | NM_001099295.1 | 2.06 |
| 6940100 | ILMN_206644 | 4930507H06Rik | AK076846 | 2.07 |
| 6270465 | ILMN_218742 | Rasgrf1 | NM_011245.1 | 2.07 |
| 2450291 | ILMN_252268 | Gm553 | XM_919936.2 | 2.07 |
| 1580598 | ILMN_201874 | 4921514L11Rik | AK014897 | 2.07 |
| 1980072 | ILMN_228494 | Sec23a | NM_009147.2 | 2.07 |
| 2340292 | ILMN_189879 | 2310058J06Rik | NM_133784 | 2.07 |
| 150128 | ILMN_213294 | Hip2 | NM_016786.2 | 2.08 |
| 5290484 | ILMN_249359 | 4930430E16Rik | NM_028672.1 | 2.09 |
| 4010037 | ILMN_215968 | Dym | NM_027727.2 | 2.09 |
| 6110750 | ILMN_201536 | Snn | NM_009223.1 | 2.09 |
| 5390440 | ILMN_236615 | Znf512b | XM_977462.2 | 2.09 |
| 3710131 | ILMN_209312 | Col20a1 | XM_181390.5 | 2.09 |
| 270762 | ILMN_204059 | Synj2 | AK038038 | 2.09 |
| 1240402 | ILMN_206985 | 4933414I19Rik | AK077156 | 2.10 |
| 2850440 | ILMN_219643 | Herpud2 | NM_020586.1 | 2.10 |
| 7560091 | ILMN_235230 | Kbtbd11 | XM_486083.4 | 2.10 |
| 5720647 | ILMN_253654 | Rsnl2 | XM_981706.1 | 2.10 |
| 3390341 | ILMN_248802 | Tmem87a | NM_173734.2 | 2.11 |
| 2680605 | ILMN_214078 | Chl1 | NM_007697.1 | 2.11 |
| 5340341 | ILMN_197106 | LOC381328 | XM_355283.1 | 2.11 |
| 4230164 | ILMN_196346 | Defcr20 | NM_183268.3 | 2.11 |
| 2690075 | ILMN_208731 | Gstcd | NM_026231.2 | 2.11 |
| 150093 | ILMN_236359 | Gm136 | NM_001033255.1 | 2.12 |
| 990064 | ILMN_221477 | Ppt1 | NM_008917.2 | 2.12 |
| 4150397 | ILMN_231393 | Hk1 | NM_010438.2 | 2.13 |
| 380451 | ILMN_215505 | Ubxn11 | NM_026257.2 | 2.13 |
| 1300674 | ILMN_216959 | Mprip | NM_201245.2 | 2.13 |
| 1010204 | ILMN_237944 | Wwtr1 | NM_133784.2 | 2.13 |
| 7650064 | ILMN_253735 | Saal1 | XM_620495.3 | 2.13 |
| 7000097 | ILMN_315139 | LOC100044257 | XM_001471785.1 | 2.14 |
| 870619 | ILMN_216574 | Lrp11 | NM_172784.2 | 2.14 |
| 4150373 | ILMN_221745 | 4.93E+23 | XM_483992 | 2.14 |
| 3610682 | ILMN_311681 | 9630041A04Rik | XR_035282.1 | 2.14 |
| 1500139 | ILMN_212623 | Klk1b8 | NM_008457.2 | 2.14 |
| 2340474 | ILMN_248075 | Ppapdc2 | NM_028922.2 | 2.14 |
| 5960026 | ILMN_220774 | D6Mm5e | NM_033079.1 | 2.14 |
| 5720553 | ILMN_220327 | Kctd11 | NM_153143.3 | 2.15 |
| 5550356 | ILMN_257640 | Gtf2ird1 | NM_001081462.1 | 2.15 |
| 1090487 | ILMN_211583 | Grin2b | NM_008171.2 | 2.15 |
| 1090546 | ILMN_221060 | Spata3 | NM_027029.1 | 2.15 |
| 3290075 | ILMN_313498 | LOC100038857 | XM_001471626.1 | 2.15 |
| 3180056 | ILMN_209080 | 1700092C17Rik | NM_183102.1 | 2.16 |
| 5820193 | ILMN_216759 | D2Ertd750e | NM_026412.2 | 2.17 |
| 580224 | ILMN_239841 | 4933402N03Rik | NM_173409.4 | 2.17 |
| 620349 | ILMN_218383 | Pemt | NM_008819.2 | 2.17 |
| 5870441 | ILMN_209820 | Cyhr1 | NM_019396.1 | 2.17 |
| 6900008 | ILMN_213435 | Myak | AF071071 | 2.17 |
| 6110463 | ILMN_232863 | Spatc1 | NM_028852.1 | 2.19 |
| 2760138 | ILMN_244736 | Akap4 | NM_001042542.1 | 2.19 |
| 1980731 | ILMN_316144 | LOC100047833 | XM_001479242.1 | 2.19 |
| 2760452 | ILMN_209330 | Larp6 | NM_026235.4 | 2.19 |
| 4200343 | ILMN_250798 | Slc39a12 | NM_001012305.1 | 2.19 |
| 430296 | ILMN_223191 | Mpped1 | NM_172610.1 | 2.20 |
| 7040546 | ILMN_242264 | Gm962 | NM_001033448.1 | 2.20 |
| 2320152 | ILMN_214543 | Ankmy1 | NM_172850.1 | 2.20 |
| 5080687 | ILMN_184752 | Zfp317 | NM_172918.3 | 2.20 |
| 1710537 | ILMN_198227 | LOC268939 | XM_110186.2 | 2.20 |
| 4040170 | ILMN_253550 | Gm347 | NM_001005420.1 | 2.20 |
| 290022 | ILMN_213345 | Lrrfip2 | XM_284541.1 | 2.21 |
| 7000014 | ILMN_313904 | LOC100046792 | XM_001476822.1 | 2.21 |
| 4290739 | ILMN_201559 | Plekhk1 | NM_133244.2 | 2.21 |
| 2370408 | ILMN_211448 | 2310061J03Rik | XM_148411.1 | 2.21 |
| 2000711 | ILMN_324925 | 1700003H04Rik | XR_034486.1 | 2.22 |
| 5270367 | ILMN_317186 | LOC100046746 | XM_001476743.1 | 2.22 |
| 1690242 | ILMN_219483 | Thsd7b | NM_172485.2 | 2.22 |
| 6350538 | ILMN_247528 | 4930538K18Rik | NM_029198.3 | 2.22 |
| 4610402 | ILMN_202326 | 4930453L07Rik | AK015455 | 2.22 |
| 60630 | ILMN_218122 | 4921517D21Rik | NM_026338.2 | 2.22 |
| 5720373 | ILMN_241119 | Gpr113 | NM_001014394.2 | 2.22 |
| 2690343 | ILMN_234927 | Grm7 | NM_177328.3 | 2.22 |
| 2370221 | ILMN_211899 | Tcp10a | NM_009340.1 | 2.22 |
| 3870347 | ILMN_188417 | Srpk2 | NM_009274 | 2.23 |
| 630113 | ILMN_211899 | Tcp10a | NM_009340.1 | 2.23 |
| 6980121 | ILMN_214543 | Ankmy1 | NM_172850.2 | 2.23 |
| 3610491 | ILMN_217746 | Rffl | NM_026097.2 | 2.24 |
| 5690253 | ILMN_202385 | 4930529I22Rik | AK015936 | 2.24 |
| 1450386 | ILMN_209168 | Iqcf3 | NM_026645.2 | 2.24 |
| 730068 | ILMN_202703 | 4930405K06Rik | AK019566 | 2.25 |
| 3130246 | ILMN_233310 | Snurf | NM_033174.2 | 2.26 |
| 2140307 | ILMN_231998 | Dnajc28 | NM_138664.2 | 2.26 |
| 6130497 | ILMN_197167 | LOC381345 | XM_355308.1 | 2.26 |
| 6560543 | ILMN_217050 | Lypla1 | NM_008866.2 | 2.26 |
| 2320452 | ILMN_212926 | BC003885 | NM_198609.2 | 2.27 |
| 2940753 | ILMN_188548 | 1700127F24Rik |  | 2.27 |
| 5860349 | ILMN_184900 | 4933433J03Rik |  | 2.27 |
| 3120168 | ILMN_259161 | Gm614 | NM_001033362.1 | 2.28 |
| 5390504 | ILMN_257635 | Lrrc8b | NM_001033550.1 | 2.28 |
| 6480168 | ILMN_212758 | Csnk1g2 | NM_134002.1 | 2.28 |
| 3450594 | ILMN_202572 | 1700112M01Rik | AK007184 | 2.29 |
| 5670373 | ILMN_209820 | Cyhr1 | NM_019396.2 | 2.29 |
| 610176 | ILMN_257493 | Pdzk1 | NM_021517.1 | 2.29 |
| 3130121 | ILMN_217418 | Agpat2 | NM_026212.1 | 2.29 |
| 10364 | ILMN_213154 | Reep6 | NM_139292.1 | 2.29 |
| 460097 | ILMN_207322 | 4930471I01Rik | AK076802 | 2.33 |
| 7320187 | ILMN_218539 | Dcp1a | NM_133761.3 | 2.33 |
| 3310398 | ILMN_220597 | Ttc24 | NM_172526.1 | 2.33 |
| 5670681 | ILMN_216009 | 4933421B21Rik | XM_130851.2 | 2.34 |
| 620326 | ILMN_215983 | Socs7 | NM_138657.3 | 2.34 |
| 3780343 | ILMN_215983 | Socs7 | NM_138657.3 | 2.35 |
| 3130139 | ILMN_259008 | Dhrsx | NM_001033326.2 | 2.35 |
| 6900279 | ILMN_221861 | Ubl4b | NM_026261.2 | 2.36 |
| 1190600 | ILMN_247528 | 4930538K18Rik | NM_029198.1 | 2.36 |
| 6770086 | ILMN_259161 | Gm614 | NM_001033362.2 | 2.36 |
| 5360193 | ILMN_242264 | Gm962 | NM_001033448.2 | 2.37 |
| 380176 | ILMN_213715 | 1700034I23Rik | NM_028494.1 | 2.38 |
| 4760487 | ILMN_220540 | Nubp2 | NM_011956.2 | 2.38 |
| 2690491 | ILMN_209820 | Cyhr1 | NM_019396.1 | 2.38 |
| 3170619 | ILMN_187856 | Ibrdc3 | XM_204030 | 2.39 |
| 3780554 | ILMN_215070 | Cdrt4 | NM_025496.1 | 2.39 |
| 5390193 | ILMN_215538 | Cd59b | NM_181858.1 | 2.39 |
| 4200592 | ILMN_211739 | Mical3 | NM_153396.1 | 2.39 |
| 5860288 | ILMN_225283 | Paqr5 | NM_028748.2 | 2.40 |
| 620079 | ILMN_230470 | Kremen1 | NM_032396.3 | 2.43 |
| 6560546 | ILMN_214033 | Fez1 | NM_183171.1 | 2.44 |
| 6480754 | ILMN_248076 | 1700011F03Rik | NM_028825.2 | 2.44 |
| 5550725 | ILMN_319033 | LOC100043347 | XM_001480344.1 | 2.44 |
| 50278 | ILMN_184752 | Zfp317 | NM_172918.3 | 2.45 |
| 4220176 | ILMN_202728 | 1700091C19Rik | AK018918 | 2.45 |
| 5550670 | ILMN_247258 | 1700008G05Rik | NM_029310.1 | 2.46 |
| 7560136 | ILMN_208976 | Clmn | NM_053155.2 | 2.46 |
| 6220364 | ILMN_323613 | LOC100045280 | XM_001473988.1 | 2.47 |
| 2370128 | ILMN_209547 | Lasp1 | NM_010688.4 | 2.47 |
| 4210243 | ILMN_202321 | 5430404L10Rik | AK015426 | 2.47 |
| 3850112 | ILMN_210112 | Dnahc8 | NM_013811.3 | 2.48 |
| 5490639 | ILMN_216311 | Oxct2b | NM_181859.2 | 2.48 |
| 3390097 | ILMN_213384 | Nrxn1 | NM_020252.2 | 2.49 |
| 6480468 | ILMN_248475 | Ccdc57 | NM_027745.1 | 2.49 |
| 2900615 | ILMN_216541 | Efcab1 | NM_025769.1 | 2.49 |
| 7570671 | ILMN_219370 | Fam20b | NM_145413.4 | 2.51 |
| 2710019 | ILMN_213239 | Mobkl2b | NM_178061.4 | 2.51 |
| 6760689 | ILMN_208661 | 4933406G16Rik | XM_488588 | 2.51 |
| 2030136 | ILMN_198842 | LOC210156 | XM_145452.2 | 2.52 |
| 610324 | ILMN_218570 | Cd3d | NM_013487.1 | 2.53 |
| 4540463 | ILMN_184131 | 4930573C08Rik |  | 2.54 |
| 2750544 | ILMN_251450 | 1700080E11Rik | NM_028562.1 | 2.55 |
| 1110092 | ILMN_219083 | Mettl3 | NM_019721.2 | 2.55 |
| 2970066 | ILMN_201536 | Snn | NM_009223.2 | 2.56 |
| 6020142 | ILMN_214623 | Tssk6 | NM_032004.1 | 2.56 |
| 2030397 | ILMN_228148 | Tmod4 | NM_016712.2 | 2.57 |
| 1240195 | ILMN_211899 | Tcp10a | NM_009340.1 | 2.57 |
| 5390181 | ILMN_253594 | 5031439G07Rik | NM_001033273.2 | 2.58 |
| 1980209 | ILMN_210831 | Actg2 | NM_009610.1 | 2.58 |
| 5560196 | ILMN_202358 | 4930500N06Rik | AK015671 | 2.59 |
| 5820500 | ILMN_209525 | Pdxdc1 | NM_053181.2 | 2.59 |
| 5390543 | ILMN_208731 | Gstcd | NM_026231.2 | 2.60 |
| 2100333 | ILMN_215482 | Lrrc57 | NM_025657.2 | 2.61 |
| 6180259 | ILMN_240650 | EG654453 | NM_001039119.1 | 2.62 |
| 3310730 | ILMN_219127 | 1700125F08Rik | XM_488557 | 2.62 |
| 2070202 | ILMN_211458 | 4932418E24Rik | NM_177841.3 | 2.64 |
| 870594 | ILMN_310517 | 1700120B22Rik | XM_001472938.1 | 2.64 |
| 580746 | ILMN_218742 | Rasgrf1 | NM_011245.2 | 2.64 |
| 2360070 | ILMN_194386 | Tmc5 | NM_028930.2 | 2.64 |
| 2570192 | ILMN_254798 | Prkcbp1 | NM_027230.3 | 2.65 |
| 4850020 | ILMN_315002 | Skp2 | NM_145468.1 | 2.66 |
| 5420039 | ILMN_209381 | A830021M18 | XM_488740 | 2.66 |
| 3180553 | ILMN_214487 | Dusp15 | NM_145744.2 | 2.67 |
| 770053 | ILMN_312362 | LOC100044756 | XM_001472989.1 | 2.67 |
| 630025 | ILMN_261018 | Mlkl | NM_029005.1 | 2.68 |
| 2810161 | ILMN_211643 | Shank2 | XM_146170.2 | 2.68 |
| 5050612 | ILMN_224041 | Pdzk1 | NM_021517 | 2.69 |
| 3120373 | ILMN_252294 | Vps36 | NM_027338.1 | 2.70 |
| 3450243 | ILMN_315002 | Skp2 | NM_145468.1 | 2.71 |
| 5420368 | ILMN_214198 | Bmi1 | NM_007552.4 | 2.71 |
| 2470671 | ILMN_202861 | Pdzk1 | AK029752 | 2.71 |
| 1660739 | ILMN_210472 | Dak | NM_145496.1 | 2.73 |
| 7400162 | ILMN_206635 | 4930402I24Rik | AK076652 | 2.73 |
| 1110255 | ILMN_215649 | Rap2ip | NM_016759.1 | 2.75 |
| 5340403 | ILMN_189726 | 4933413G19Rik | XM_132869 | 2.75 |
| 2190524 | ILMN_316567 | LOC100048703 | XM_001480914.1 | 2.75 |
| 990095 | ILMN_215845 | BC055111 | NM_183182.2 | 2.76 |
| 1170014 | ILMN_239476 | LOC668433 | XM_001003627.1 | 2.76 |
| 5270564 | ILMN_211711 | 1700016H13Rik | XM_132216.7 | 2.76 |
| 6960327 | ILMN_247034 | 1700020A23Rik | XM_918226.2 | 2.77 |
| 4040154 | ILMN_184645 | C230098O21Rik |  | 2.79 |
| 4920367 | ILMN_197218 | LOC381362 | XM_355328.1 | 2.80 |
| 3840181 | ILMN_201044 | LOC381963 | XM_355995.1 | 2.81 |
| 4250148 | ILMN_228148 | Tmod4 | NM_016712.2 | 2.82 |
| 7550441 | ILMN_259837 | Ihpk2 | NM_029634.1 | 2.82 |
| 4560626 | ILMN_216100 | Glo1 | NM_025374.2 | 2.84 |
| 6130368 | ILMN_327461 | LOC100044299 | XM_001471746.1 | 2.85 |
| 6450475 | ILMN_196486 | 2410072D24Rik | XM_357002.1 | 2.85 |
| 4060343 | ILMN_211275 | Ubqlnl | NM_198624.3 | 2.87 |
| 6020224 | ILMN_221311 | Il4i1 | NM_010215.2 | 2.88 |
| 1510392 | ILMN_216662 | Phospho1 | NM_153104.2 | 2.89 |
| 4610193 | ILMN_318408 | LOC100046393 | XM_001476699.1 | 2.90 |
| 2230221 | ILMN_222473 | Ppid | NM_026352.2 | 2.90 |
| 6270471 | ILMN_314701 | 1700009J07Rik | XR_035305.1 | 2.91 |
| 4060437 | ILMN_323640 | LOC100044103 | XM_001472411.1 | 2.92 |
| 4780129 | ILMN_227423 | Clec4g | NM_029465.2 | 2.93 |
| 630504 | ILMN_238101 | Rbm28 | NM_026650.1 | 2.94 |
| 460300 | ILMN_210174 | 4933400C05Rik | NM_177801.2 | 2.96 |
| 6350209 | ILMN_255002 | EG384639 | NM_001013781.1 | 2.97 |
| 2690102 | ILMN_216387 | Fntb | NM_145927.2 | 2.98 |
| 5860097 | ILMN_196486 | Psma8 | XM_357002.5 | 2.99 |
| 430400 | ILMN_232012 | 1700058C13Rik | NM_028528.2 | 3.00 |
| 3190246 | ILMN_214987 | Slc13a4 | NM_172892.1 | 3.01 |
| 1940373 | ILMN_219609 | Oxct2a | NM_022033.2 | 3.01 |
| 5900343 | ILMN_217017 | 1700012A03Rik | NM_029587.2 | 3.01 |
| 4890768 | ILMN_215320 | 1700067K01Rik | NM_183097.1 | 3.01 |
| 2680066 | ILMN_326750 | AI507611 | XM_001477461.1 | 3.02 |
| 2810369 | ILMN_192397 | 0710001D07Rik |  | 3.03 |
| 1170142 | ILMN_201900 | 4932442I06Rik | AK016813 | 3.03 |
| 6510575 | ILMN_257307 | LOC675899 | XM_985882.1 | 3.05 |
| 7610414 | ILMN_194005 | 4930486I03Rik |  | 3.07 |
| 3060110 | ILMN_219969 | Cstl1 | NM_177655.3 | 3.10 |
| 2360592 | ILMN_191668 | 1700052O22Rik |  | 3.12 |
| 4490253 | ILMN_210568 | Klb | NM_031180.2 | 3.13 |
| 5290204 | ILMN_257192 | Ift140 | NM_134126.2 | 3.13 |
| 430639 | ILMN_253474 | Dydc2 | NM_027717.1 | 3.16 |
| 6350161 | ILMN_257192 | Ift140 | NM_134126.2 | 3.16 |
| 6960037 | ILMN_214236 | Cyp2a12 | NM_133657.1 | 3.19 |
| 2490605 | ILMN_186598 | Dhrsx | XM_135485 | 3.21 |
| 240286 | ILMN_216759 | D2Ertd750e | NM_026412.1 | 3.23 |
| 7100053 | ILMN_218806 | 1700011H14Rik | NM_025956.3 | 3.27 |
| 6620292 | ILMN_222190 | 4930549C01Rik | NM_026300.2 | 3.27 |
| 1170369 | ILMN_220204 | 4930474M22Rik | NM_183107.1 | 3.29 |
| 4900358 | ILMN_213282 | Npb | NM_153288.3 | 3.30 |
| 2340066 | ILMN_257615 | Vars2 | NM_175137.3 | 3.32 |
| 6660114 | ILMN_216396 | Spo11 | NM_012046.1 | 3.33 |
| 780039 | ILMN_226714 | 4930455F16Rik | XM_986656.1 | 3.38 |
| 3290164 | ILMN_311580 | LOC622994 | XR_031862.1 | 3.41 |
| 6280435 | ILMN_199348 | Gm77 | XM_359331.5 | 3.42 |
| 7400408 | ILMN_254798 | Prkcbp1 | NM_027230.3 | 3.43 |
| 2350603 | ILMN_201921 | Ncam | AK017085 | 3.45 |
| 3940598 | ILMN_219371 | 4933428D01Rik | XM_127662.2 | 3.85 |
| 5290711 | ILMN_210229 | Car2 | NM_009801.3 | 3.89 |
| 2360564 | ILMN_206634 | 4921505L17Rik | AK076554 | 3.95 |
| 1510435 | ILMN_254798 | Prkcbp1 | NM_027230.3 | 4.10 |
| 6520288 | ILMN_208782 | Nqo2 | NM_020282.2 | 4.19 |
| 1980435 | ILMN_227937 | 1700023A16Rik | XM_909859.2 | 4.20 |
| 650338 | ILMN_219728 | Adam21 | NM_020330.4 | 4.33 |
| 270187 | ILMN_208782 | Nqo2 | NM_020282.2 | 4.42 |
| 6110014 | ILMN_208782 | Nqo2 | NM_020282.2 | 4.49 |
| 5870367 | ILMN_319803 | LOC666403 | XR_034389.1 | 4.64 |
| 4850136 | ILMN_316949 | LOC100047899 | XM_001479088.1 | 4.69 |
| 6250408 | ILMN_196588 | Defcr-rs1 | NM_007844.1 | 4.92 |
| 2340546 | ILMN_207466 | Chn1 | NM_029716.2 | 4.99 |
| 2650300 | ILMN_217434 | Gfer | NM_023040.3 | 5.02 |
| 3830678 | ILMN_217772 | Fcgr3 | NM_010188.4 | 5.06 |
| 7000315 | ILMN_207416 | BC002216 | AK089229 | 7.91 |
| 5570736 | ILMN_221469 | Cgnl1 | NM_026599.4 | 8.21 |

Differentially expressed genes that are associated with functional clusters described in Table 2 are shown in blue texts.
