# Supplementary material for: RBM5 Is a Male Germ Cell Splicing Factor and Is Required for Spermatid Differentiation and Male Fertility
Source: PLoS Genet. 2013 Jul 25;9(7):e1003628. doi: 10.1371/journal.pgen.1003628 (PMC3723494; doi:10.1371/journal.pgen.1003628)
Supplement: Table S2 — (DOC) [file pgen.1003628.s003.doc]

**Supplementary Table S2: RBM5 interacting proteins in round spermatids identified using immunoprecipitation and mass spectrometry analysis**

Band 1 (~110 kDa)

| **Symbol** | **Name** | **Accession** | **Score / No. matched peptides** |
| --- | --- | --- | --- |
| *SFPQ | Splicing factor, praline- and glutamine-rich | Q8VIJ6 | 317/6 |
| IPO5 | Importin 5 | Q8BKC5 | 170/4 |
| HYOU1 | Hypoxia up-regulated protein 1 | Q9JKR6 | 125/2 |
| *MATR3 | Matrin 3 | Q8K310 | 90/2 |
| PSMD1 | 26S proteasome non-ATPase regulatory subunit 1 | Q3TXS7 | 67/2 |
| NASP | Nuclear autoantigenic sperm protein | Q99MD9 | 65/2 |
| TERA (VCP) | Transitional endoplasmic reticulum ATPase | Q01853 | 64/4 |
| ACE | Angiotensin-converting enzyme | P09470 | 56/2 |
| LRRC50 | Leucine-rich repeat-containing protein 50 | AAH50751 | 52/2 |
| *hnRNP UL1 | Heterogeneous nuclear ribonucleoprotein U-like protein 1 | Q8VDM6 | 45/3 |

**Band 2 (~90 kDa)**

| **Symbol** | **Name** | **Accession** | **Score / No. matched peptides** |
| --- | --- | --- | --- |
| PABP1 | Polyadenylate-binding protein 1 | P29341 | 587/19 |
| TERA | Transitional endoplasmic reticulum ATPase | Q01853 | 284/8 |
| *hnRNP M | Heterogeneous nuclear ribonucleoprotein M | Q9D0E1 | 178/4 |
| *PSIP1 | PC4 and SFRS1-interacting protein | Q99JF8 | 101/3 |
| DDX4 (VASA) | Probable ATP-dependent RNA helicase DDX4 | Q61496 | 46/1 |
| *DDX5 | Probable ATP-dependent RNA helicase DDX5 | Q61656 | 41/1 |

**Band 3 (~70 kDa)**

| **Symbol** | **Name** | **Accession** | **Score / No. matched peptides** |
| --- | --- | --- | --- |
| TCPE | T-complex protein 1 subunit epsilon | P80316 | 340/10 |
| *hnRNP K | Heterogeneous nuclear ribonucleoprotein K | P61979 | 337/9 |
| TCPG | T-complex protein 1 subunit gamma | P80316 | 331/13 |
| ODP2 | Dihydrolipoyllysine-residue acetyltransferase component of pyruvate dehydrogenase complex, mitochondrial | Q8BMF4 | 311/12 |
| MYEF2 | Myelin expression factor 2 | Q8C854 | 226/6 |
| TCPZ | T-complex protein 1 subunit zeta | P80317 | 222/7 |
| TCPA | T-complex protein 1 subunit alpha | P11983 | 204/7 |
| PSPC1 | Paraspeckle component 1 | Q8R326 | 164/5 |
| GRP78 | 78 kDa glucose-regulated protein | P20029 | 132/2 |
| DHAK | Bifunctional ATP-dependent dihydroxyacetone kinase/FAD-AMP lyase (cyclizing) | Q8VC30 | 110/2 |
| PDIA3 | Protein disulfide-isomerase A3 | P27773 | 96/5 |
| TCPQ | T-complex protein 1 subunit theta | P42932 | 95/4 |
| CACP | Carnitine O-acetyltransferase | P47934 | 73/4 |
| PSMD3 | 26S proteasome non-ATPase regulatory subunit 3 | P14685 | 63/2 |

**Band 4 (~30 kDa)**

| **Symbol** | **Name** | **Accession** | **Score / No. matched peptides** |
| --- | --- | --- | --- |
| *hnRNP A2/B1 | Heterogeneous nuclear ribonucleoproteins A2/B1 | O88569 | 370/19 |
| LDHC | L-lactate dehydrogenase C chain | P00342 | 266/13 |
| RS3A | 40S ribosomal protein S3a | P97351 | 192/6 |
| ADT4 | ADP/ATP translocase | Q3V132 | 110/4 |
| VDAC2 | Voltage-dependent anion-selective channel protein 2 | AAC13321 | 105/2 |
| RSPH9 | Radial spoke head protein 9 homolog | Q9D9V4 | 84/2 |
| *U1A (SNRPA) | U1 small nuclear ribonucleoprotein A | Q62189 | 71/2 |
| MTCH2 | Mitochondrial carrier homolog 2 | Q791V5 | 71/3 |
| ELAV1 (HuR) | Embryonic lethal abnormal vision-like protein 1 | P70372 | 62/2 |
| *SFRS1 (ASF/SF2) | Splicing factor, arginine/serine-rich 1 | Q6PDM2 | 53/2 |

Accession numbers: UniProtKB/Swiss-Prot. * indicates proteins with splicing-related function
