# Supplementary material for: RBM5 Is a Male Germ Cell Splicing Factor and Is Required for Spermatid Differentiation and Male Fertility
Source: PLoS Genet. 2013 Jul 25;9(7):e1003628. doi: 10.1371/journal.pgen.1003628 (PMC3723494; doi:10.1371/journal.pgen.1003628)
Supplement: Table S3 — (DOCX) [file pgen.1003628.s004.docx]

**Supplementary Table S3: Primers used for genotyping and RT-PCR analysis**

| **Primer name** | **Sequence (5’🡪3’)** | | **Detail** |
| --- | --- | --- | --- |
| **Genotyping of the *Rbm5^MU^* mouse line** | | | |
| Rbm5-WT | GAAGGTGACCAAGTTCATGCTGTGTCTGTTTGTCTTTTATGAGGC | | C (WT, wild-type) and G (MU, mutation) are underlined. |
| Rbm5-MU | GAAGGTCGGAGTCAACGGATTTTGTGTCTGTTTGTCTTTTATGAGGG | |  |
| Rbm5-Rev | CCCTATCTCCCTATGCTTCCTT | |  |
| **Genotyping of the *Rbm5* gene-trap mouse line** | | | |
| Ex1-Rbm5-Fw | CTCCTGCTTTGTTCCCTCTG | | Ex1-Rbm5-Fw + Int1-Rbm5-Rev primers amplify the WT allele (799 bp).  Ex1-Rbm5-Fw + Neo-IN21- amplify the gene-trap allele (983 bp). |
| Int1-Rbm5-Rev | CCAAACCTTGACTCGGCTTA | |  |
| Neo-IN21-Fw | CAAGGACCTGAAATGACCCTGTG | |  |
| **Detection of splicing defects** | | | |
| St5-Ex2-Fw | | ATGACCATGACTGCCAACAA | Amplify *St5* cDNA (ENSMUST00000077909) from exons 2 – 5 |
| St5-Ex5-Rev | | TCCAGCTCTCCGGTTCTTTA |  |
| Asb1-Ex1-Fw | | TGACAGCGTTGCTCTCTGAC | Amplify *Asb1* cDNA (ENSMUST00000027538) from exons 1 – 5 |
| Asb1-Ex5-Rev | | CAGATGCAGTCGGTATTTGC |  |
| Pla2g10-Ex1-Fw | | CCTCTGTGACGGGCAATAAT | Amplify *Pla2g10* cDNA (ENSMUST00000023364) from exons 1 – 4 |
| Pla2g10-Ex4-Rev | | TTGGCATTTGTTCTCTGCTG |  |
| Kif17-Ex10-Fw | | AACCTGAGAGTGGCTGAGGA | Amplify *Kif17* cDNA (ENSMUST00000030539) from exons 10 – 15 |
| Kif17-Ex15-Rev | | TTGGAGAAGGGGATGTCAAG |  |
| Anks3-Ex2Fw | | GAGCCGGAGCTCCTGAAG | Amplify *Anks3* cDNA (ENSMUSG00000022515) from exons 2 – 17 |
| Anks3-Ex17Rev | | CACCCCAGACCTTGTCTGTAA |  |
| Rangap1-Ex3Fw | | ATGGCCTCTGAAGACATTGC | Amplify *Rangap1* cDNA ENSMUSG00000022391 from exons 3 – 17 |
| Rangap1-Ex17Rev | | GGGCAAAGGAGCAGGTTT |  |
| Nxf1-Ex1Fw | | CCCCTGTACCTGCCTGTG | Amplify *Nxf1* cDNA (ENSMUST00000010241) from exons 1 – 7 |
| Nxf1-Ex7Rev | | TGAGGTCAAGTGCTTGTTGG |  |
| Cftr-Ex17Fw | | GTGGGAGTGGCTGACACTTT | Amplify *Cftr* cDNA (ENSMUST00000045706) from exons 17 – 27 |
| Cftr-Ex27Rev | | GCCTGAAGGGAGTCGTACTG |  |
